# Copper(I) Catalyzed Decarboxylative Synthesis of Diareno[*a*,*e*]cyclooctatetraenes

**DOI:** 10.1021/acs.joc.2c00286

**Published:** 2022-05-19

**Authors:** Magdalena Tasić, Albert Ruiz-Soriano, Daniel Strand

**Affiliations:** Centre for Analysis and Synthesis, Department of Chemistry, Lund University, Box 124, SE-221 00 Lund, Sweden

## Abstract

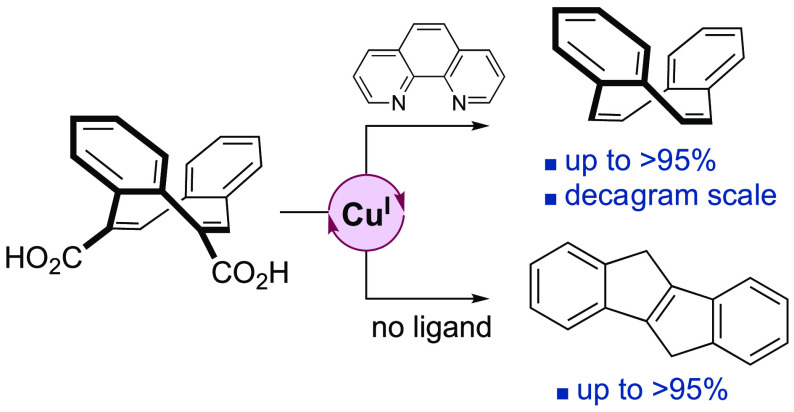

Diareno[*a*,*e*]cyclooctatetraenes find widespread applications as building blocks, ligands, and responsive cores in topologically switchable materials. However, current synthetic methods to these structures suffer from low yields or operational disadvantages. Here, we describe a practical three-step approach to diareno[*a*,*e*]cyclooctatetraenes using an efficient copper(I) catalyzed double decarboxylation as the key step. The sequence relies on cheap and abundant reagents, is readily performed on scale, and is amenable also to unsymmetrical derivatives that expand the utility of this intriguing class of structures.

Diareno[*a*,*e*]cyclooctatetraenes and their derivatives comprise a remarkably useful class of functional molecules.^1,2^ Applications span from size-switchable cavities^3^ to molecular tweezers,^4,5^ materials for energy storage,^6,7^ force^8,9^ and free volume probes,^10^ adhesives,^11^ and light-emitting devices.^12−15^ Perhaps the most prominent implementations of the parent members, dibenzo[*a*,*e*]cyclooctatetraene (dbCOT, **2**)^16^ and dinaphto[*a*,*e*]cyclooctatetraene (dnCOT, **5**),^17^ are found in coordination chemistry as bis-η^2^ ligands.^18^ dbCOT was first developed as a catalyst-poison by Crabtree,^19^ but more recently, both dbCOT and dnCOT have been shown to confer both enhanced stability and superior performance when used as ligands in several catalytic systems.^20−22^ Notable examples include iridium-catalyzed allylic substitutions,^23−29^ iridium- and rhodium-catalyzed (5 + 2) cycloadditions,^17,30^ a ruthenium-catalyzed oligomerization,^31^ and rhodium-catalyzed polymerization reactions.^32^ Our interest in these structures stems from the utility of dbCOT derivatives as both topological switches in [8]annulene-based *pseudo*-conjugated materials^33^ and as efficient planar-chiral steering ligands in asymmetric catalysis.^34^ However, despite a superficial structural simplicity and utility across several areas of research, a general, efficient, and practical synthesis of diareno[*a*,*e*]cyclooctatetraenes has remained a challenge. The first synthesis of dbCOT was reported by Pechet in 1946 using copper chromite to achieve a low-yielding double decarboxylation of dicarboxylic acid **1** (Figure 1).^35^

**Figure 1:**
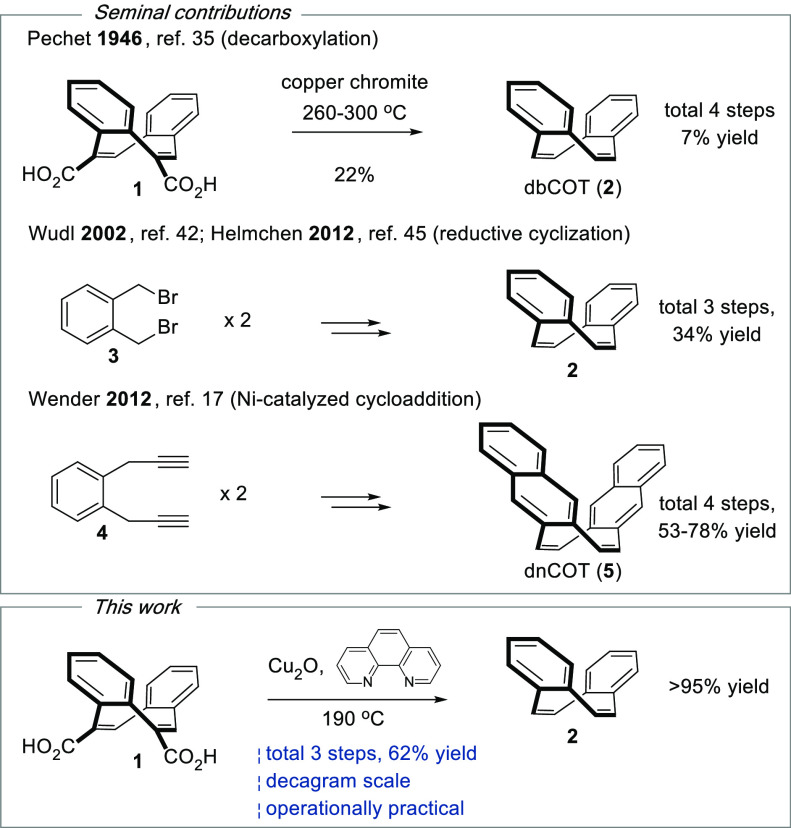
Synthetic approaches to dbCOT (**1**) and dnCOT (**5**)

Since then, alternative methods using Wittig olefinations,^36−38^ photoisomerizations,^39−41^ and ring expansion reactions^42,43^ have been developed. For dnCOT, a step-economical synthesis was developed by Wender via an imaginative nickel-catalyzed (2 + 2 + 2 + 2) cycloaddition.^17^ The critical advance that supports much of the current research on dbCOT is Fenton’s route via a reductive dimerization of dibromide **3**.^44^ This procedure was developed to a gram scale synthesis by Wudl,^42^ and the protocol was further refined by Helmchen.^45^ Unfortunately, even the latter procedure is burdened to some extent by operational disadvantages related to the sensitivity, toxicity, or reactivity of several reagents and solvents as well as a cumbersome separation of higher oligomers formed as byproducts in the dimerization of **3**. Consequently, dbCOT and homologous structures have remained prohibitively expensive for many applications.

Motivated by the limitations of current protocols and the potential of substrates with different flanking arenes, we revisited Pechet’s original approach with the aim of developing an improved protocol by exploiting recent advances in catalytic decarboxylation reactions.^46−49^ Here, we report an operationally simple, cheap, and efficient Cu_2_O/1,10-phenanthroline catalyzed double decarboxylation of **1** to produce dbCOT. Implemented in a three-step sequence starting from abundant materials, the overall yield is almost double that of previous syntheses. The method extends to the homologous dnCOT^17^ and, significantly, also to the unsymmetrical benzonaphto[*a*,*e*]cyclooctatetraene (bnCOT, **23**).^50^ A noteworthy finding is that in the absence of a donor ligand, the decarboxylation of **1** can be steered through an alternative reaction manifold to cleanly produce 5,10-dihydroindeno[2,1-*a*]indene (**6**); a useful building block in photovoltaic materials^51,52^ and a precursor to *C*_2_-symmetric 5,11-disubstituted dbCOTs with applications as ligands in asymmetric catalysis.^34^

At the outset, we thus sought an efficient and cheap catalyst system to convert dicarboxylic acid **1** to dbCOT. A number of methods for direct decarboxylation of aromatic carboxylic acids have been introduced recently,^46,47^ but aliphatic and vinylic variations are less common.^53−58^ Pleasingly, we found that copper(I) salts were able to promote the vinylic decarboxylation of **1** to produce dbCOT **2**. Djakovitch’s procedure using 20 mol % Cu(OH)_2_ as catalyst^56^ was first evaluated but gave a fair 21% yield (Table 1, entry 1).

**Table 1:**
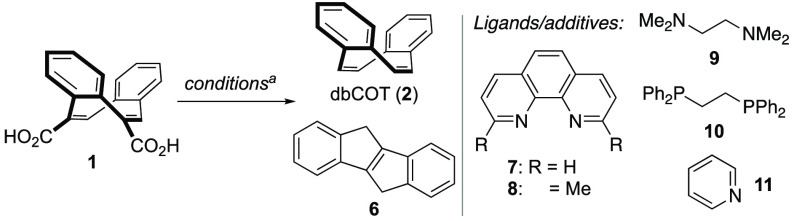
Optimization of the Decarboxylation of Dicarboxylic Acid **1**^a^

entry	catalyst (mol %)	ligand/additive (mol %)	temp (°C)	selectivity **2**:**6**	product, yield^b^ (%)
1	Cu(OH)_2_ (20)	**7** (20), **9** (100)	150	23:77	**2**, 21
2	Cu_2_O (7.5)	**7** (15), **9** (100)	190	100:0	**2**, >95^c^
3	Cu_2_O (7.5)	**7** (15)	190	100:0	**2**, >95^d^
4^e^	Cu_2_O (7.5)	**7** (15)	190	100:0	**2**, 77^c^
5	Cu_2_O (5.0)	**7** (10), **9** (100)	190	88:12	**2**, 87
6	Cu_2_O (10)	**7** (20), **9** (100)	170	92:8	**2**, 55
7	CuBr (20)	**7** (20), **9** (100)	170	51:49	**2**, 11
8	CuI (20)	**7** (20), **9** (100)	170	19:81	**2**, 1
9	Cu_2_O (10)	**8** (20), **9** (100)	170	2:98	**6**, 5
10	Cu_2_O (10)	**10** (20), **9** (100)	170	64:36	**2**, 17
11^f^	AgOAc (20)	K_2_S_2_O_8_ (500)	100	100:0	**2**, 4
12^g^	Pd(PPh_3_)_4_ (15)	**11** (120), H_2_O	50		
13	Cu_2_O (7.5)		190	0:100	**6**, >95^c^
14			190	0:100	**6**, 92^c^

a General conditions: dicarboxylic acid **1** (4.6 mM), catalyst, and additive were dissolved in *N*-methyl-2-pyrrolidone and heated to the indicated temperature under an inert atmosphere for 24 h.

b Yield of the major product as determined by ^1^H NMR spectroscopy using 1-methoxynaphthalene as an internal standard.

c Isolated yield: 100 mg scale.

d Isolated yield: 3.0 g scale.

e Reaction conducted under an air atmosphere;

f For conditions, see ref (59).

g For conditions, see ref (58).

Systematic variations of this procedure revealed that cheap copper(I) oxide (7.5 mol %) with 1,10-phenanthroline (**7**) as ligand and *N*,*N*,*N*′,*N*′-tetramethylethylenediamine (TMEDA, **9**) as additive gave dbCOT in essentially quantitative yield from **1** at elevated temperature (190 °C, entry 2). Mechanistically, such reactions catalyzed by Cu(I)/1,10-phenanthroline have been shown to proceed via formation of a Cu(I) carboxylate salt, followed by insertion into the C–C bond to trigger the loss of CO_2_.^60^ Omitting TMEDA resulted in a minor decrease in the isolated yield and simplified isolation of dbCOT by avoiding formation of emulsions during the extractive workup (entry 3). An inert atmosphere was however found necessary to preserve the efficiency of the reaction (entry 4), presumably to avoid oxidation of Cu(I) to Cu(II) species. Further optimization showed that reducing the amount of Cu_2_O below 7.5 mol % resulted in the emergence of indene **6**(51) as a side product (entry 5). This byproduct was also observed by Pechet with copper chromite as the reagent.^35^ Decreasing the temperature to 170 °C reduced the conversion by 40%, even with 10 mol % catalyst (entry 6). Moreover, copper(I) bromide and iodide were evaluated but gave significantly lower conversions and increased amounts of **6** compared to copper(I) oxide (entries 7 and 8). We also evaluated different ligands. When substituting 1,10-phenanthroline for neocuproine (**8**), dbCOT **2** was formed in trace amounts, and with 1,2-bis(diphenylphosphino)ethane (**10**) a 17% yield was obtained (entries 9 and 10). Other catalytic systems based on silver(I)^59^ and palladium(0)^58^ were evaluated but gave at most trace amounts of dbCOT (Table 1, entries 11 and 12; see also the Supporting Information for additional details of the optimization study).

Interestingly, omitting 1,10-phenanthroline led to a complete switch in the reaction manifold to give indene **6** in essentially quantitative yield (entry 13). Simply heating **1** in *N*-methyl-2-pyrrolidone (NMP) also led to formation of **6** in 92% yield but with a less clean reaction profile and incomplete conversion (4% recovered starting material after 24 h) (entry 14). In light of the faster and cleaner reaction with Cu_2_O, it seems plausible that the decarboxylation steps are facilitated by Cu(I). Clarifying the nuances of the role of Cu(I) in this mechanism, which presumably involves via radical intermediates,^61^ will require further investigations. The efficient formation of **6** from abundant **1** is noteworthy for several reasons. First, **6** finds immediate application as a building block for photovoltaic materials.^51^ Second, oxidative cleavage of **6** to diketone **12** by ozonolysis has previously been reported in 31% yield.^44^ Diketone **12** is useful as it can be straightforwardly converted to the corresponding divinyltriflate which, in turn, is a gateway compound to diverse planar chiral 5,11-disubstituted dbCOTs with applications as steering ligands in asymmetric catalysis.^34^ We found that a ruthenium(IV) oxide catalyzed oxidation of **6** to **12** provided a convenient alternative to ozonolysis that also improved the isolated yield to 73% (Scheme 1).

**Scheme 1:**
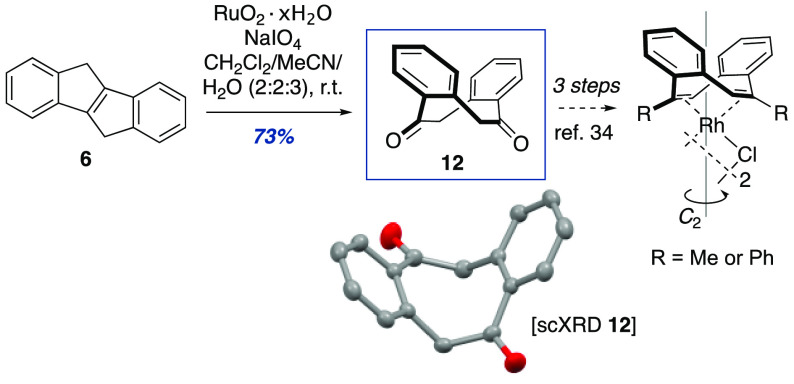
Ruthenium-Catalyzed Oxidative Cleavage of Indene **6**

With an optimized procedure for the double decarboxylation of **1** at hand, we turned to implement this reaction as part of a practical and scalable synthesis of dbCOT. To this end, we first investigated the known condensation of **13** and **14** to form dinitrile **15** (Scheme 2).^35^

**Scheme 2:**
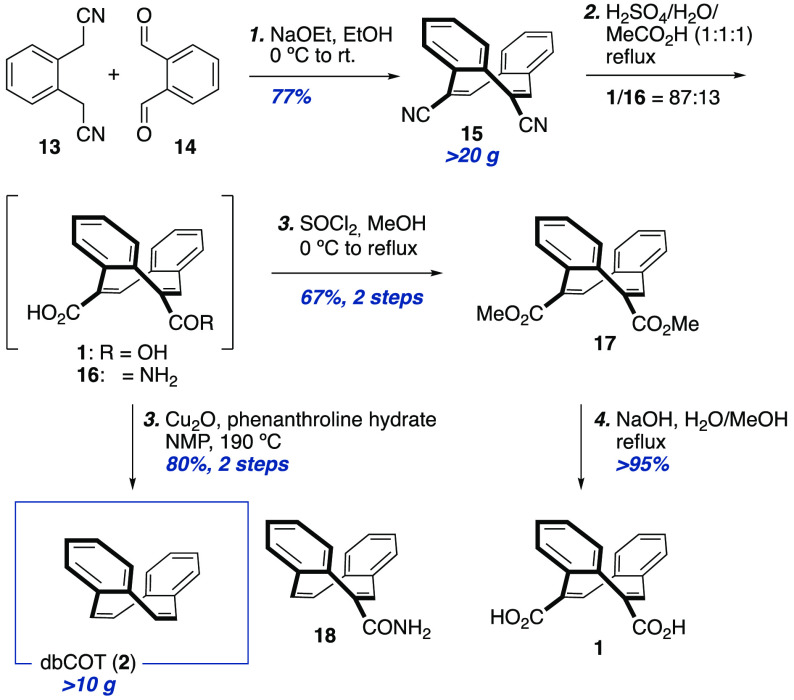
Decagram Scale Synthesis of dbCOT (**2**)

Careful optimization of Pechet’s conditions revealed that an inert atmosphere and slow addition of the base (sodium ethoxide) were beneficial factors that increased the yield from a previously reported 47% to 77%. The cyclized product **15** could be collected in a sufficiently pure form by filtration of the reaction mixture. These conditions were scaled to over 20 g with a retained efficiency. Replacing dinitrile **13** with the corresponding dimethyl ester in the condensation step was also evaluated using sodium methoxide as the base but did not provide the corresponding condensation product.

In Pechet’s procedure, dinitrile **15** was first converted to the corresponding diethyl ester using EtOH/HCl and then hydrolyzed to dicarboxylic acid **1** under basic conditions. To reduce the overall step-count and avoid the need for gaseous HCl, we sought a protocol for hydrolyzing dinitrile **15** directly to **1**. Pleasingly, hydrolysis using a mixture of sulfuric acid/water/acetic acid (1:1:1)^62^ gave dicarboxylic acid **1** together with minor amounts of monocarboxamide **16**. On a gram scale, **1** and **16** were obtained in a 95:5 ratio. When scaling the reaction linearly to 17 g, the ratio was reduced to 87:13. This difference was consistent between experiments and is likely related to an equilibrium where the ammonium ion concentration in the heated mixture is influenced by the reaction setup. Despite extensive experimentation, we were not able to identify a convenient hydrolysis method that further improved the efficiency (for details, see the Supporting Information, Table S2). Resubjection of the mixture of **1** and **16** to the hydrolysis conditions could however be used to increase the ratio to 94:6. Performing the hydrolysis using a sulfuric acid/water mixture (1:1)^35^ was evaluated but gave sluggish results above a gram scale. Because separation of **1** and **16** is cumbersome, the decarboxylation was then performed directly on the 87:13 mixture obtained from the hydrolysis step. To achieve a clean reaction, the amount of copper catalyst was increased from 7.5 to 12.5 mol %. Under these conditions, dbCOT and carboxamide **18**, formed by decarboxylation of **16**, were the only observed products. The carboxamide could be removed by suspending the crude mixture in toluene followed by filtration through a short plug of silica (washed with toluene) to give dbCOT in 80% yield over two steps from **15**. The procedure was evaluated on over a 10 g scale without loss of efficiency. The carboxamide **18** could also be recovered in 8% yield (over two steps from **15**) by a subsequent wash of the silica plug with MeOH.

The overall yield of the sequence could be improved by resubjecting the isolated carboxamide **18** to basic hydrolysis with NaOH followed by decarboxylation to produce an additional 4% of dbCOT (see the Supporting Information for experimental details), but this was not found more practical than reperforming the synthesis from commercial **13** and **14**. In addition, pure dicarboxylic acid **1** could be obtained by subjecting mixtures of **1** and **16** to SOCl_2_ in MeOH to obtain dimethyl ester **17**, which was then hydrolyzed under basic conditions to produce **1** in 67% yield over two steps.

With an efficient and scalable decarboxylative approach to dbCOT at hand, we turned to investigate the generality of the sequence by applying it also to the synthesis of dnCOT (**5**) and bnCOT (**23**) (Scheme 3).

**Scheme 3:**
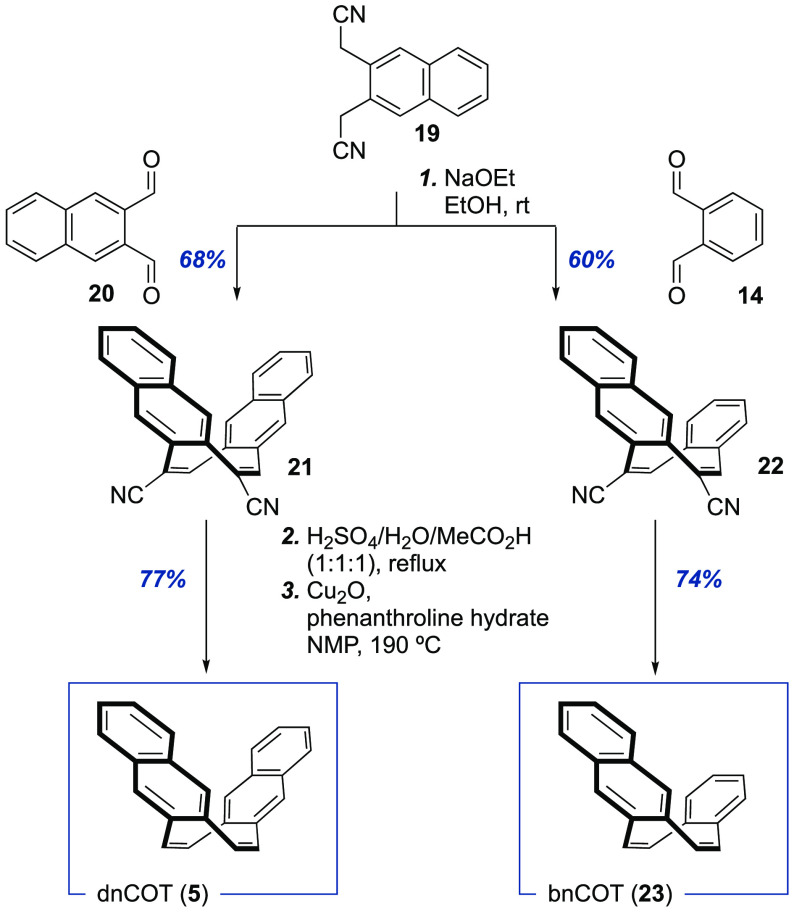
Synthesis of dnCOT (**5**) and bnCOT (**23**)

Pleasingly, both compounds were readily obtained in good yield without the need for further optimization of the reaction conditions. The overall yield of 52% in the synthesis of dnCOT is comparable to Wender’s synthesis^17^ but has a lower step-count and uses cheaper reagents. For the nonsymmetrically substituted bnCOT, a total yield of 44% represents a considerable improvement over the 4% yield achieved with previous methods.^50^

In conclusion, an efficient copper(I) catalyzed decarboxylation of vinylic dicarboxylic acids like **1** has been developed. When implemented as the key step in the syntheses of three diareno[*a*,*e*]cyclooctatetraenes, dbCOT, dnCOT, and bnCOT, the three-step sequences are characterized by cheap and benign reagents and practical procedures. The improved efficiency in the key decarboxylation step compared to Pechet’s method is likely related to the lower reaction temperatures allowed with homogeneous copper(I) catalysts and thereby avoiding a competing background reaction forming indene **6**. The described method using strong acids and elevated temperatures in the hydrolysis and decarboxylation steps, respectively, is optimized for pure hydrocarbons like dbCOT. More broadly, we anticipate that further refinements, such as mild metal-catalyzed conditions for the nitrile hydrolysis step,^63^ and redox activation of the carboxylic acid intermediates to facilitate the decarboxylation step^48,49^ will serve to further expand the utility of the approach. Such studies are under way and will be reported in due course.

## Experimental Section

### General Information

All reagents and solvents were bought from commercial suppliers and used as received unless otherwise stated. All reactions were conducted in oven-dried glassware under a nitrogen atmosphere using magnetic stirring unless otherwise stated. ^1^H and ^13^C (^1^H decoupled) NMR spectroscopy data were collected on a Bruker Avance II 400 MHz (^1^H 400 MHz; ^13^C 101 MHz) equipped with a 5 mm BBOF Z-gradient probe. Multiplicities are denoted by singlet (s), doublet (d), and multiplet (m). Broad peaks are denoted by (br). FTIR spectra were recorded on a Bruker Alpha II spectrometer and peaks denoted as strong (s), medium (m), weak (w), and broad (br). HRMS data were obtained using an ESI-QTOF mass spectrometer (Waters Xevo-G2) in positive or negative mode between *m*/*z* 50–1200, employing lockmass correction according to the manufacturer’s instructions. Thin-layer chromatography (TLC) was performed using Merck 60 F_254_ silica gel bound to aluminum plates. Purification by column chromatography was performed using the Biotage Isolera One system.

### (5*E*,11*E*)-Dibenzo[*a*,*e*][8]annulene-5,12-dicarbonitrile (**15**)

To a stirred solution of dinitrile **13** (20.0 g, 128 mmol) and dialdehyde **14** (20.0 g, 149 mmol) in ethanol (450 mL) at 0 °C was added sodium ethoxide (21.0 wt % in ethanol, 24.0 mL, 64.3 mmol) dropwise over 30 min. The resulting orange mixture was warmed up to room temperature, stirred for 3 h, and then cooled using an ice–water bath. The formed precipitate was collected by filtration, washed with cold ethanol (300 mL), and dried under reduced pressure to afford dinitrile **15**. Yield: 25.2 g (77%). Isolated as a pale brown amorphous solid, >95% pure by NMR spectroscopy and a single spot by TLC. *R*_*f*_: 0.66 (CH_2_Cl_2_). ^1^H NMR (CDCl_3_, 400 MHz): δ 7.61 (s, 2H), 7.44–7.37 (m, 4H), 7.37–7.31 (m, 2H), 7.19–7.11 (m, 2H) ppm. ^13^C{^1^H} NMR (CDCl_3_, 101 MHz): δ 147.2, 133.5, 132.5, 130.1, 129.5, 129.4, 128.9, 118.7, 117.5 ppm. FTIR (CH_2_Cl_2_ film): 3057 (w), 3031 (w), 2220 (m), 1628 (w), 1491 (w), 1430 (w), 1364 (w) cm^–1^. HRMS (ESI-TOF) *m*/*z*: [M]^+^ calcd for C_18_H_10_N_2_ 254.0844; found 254.0831.

### (5*Z*,11*Z*)-Dibenzo[*a*,*e*][8]annulene (dbCOT, **2**)

To dinitrile **15** (17.4 g, 68.4 mmol) was added glacial acetic acid (130 mL), water (130 mL), and concentrated sulfuric acid (130 mL). The resulting reaction mixture was heated to reflux for 30 h, then cooled down to room temperature, and poured onto ice (200 mL). The brown precipitate was collected by filtration and the filtrate extracted with diethyl ether (3 × 200 mL). The combined organic layers were washed with brine (200 mL), passed through a phase separator, and concentrated under reduced pressure. The remaining solid residue was combined with the previously collected precipitate and dried under reduced pressure to furnish an 87:13 mixture of hydrolysis products **1** and **16** as a brown solid (19.6 g). This solid was charged in a round-bottom flask together with Cu_2_O (1.20 g, 8.39 mmol) and 1,10-phenanthroline monohydrate (2.00 g, 10.1 mmol), and *N*-methyl-2-pyrrolidone (490 mL) was added. The resulting red solution was heated to 190 °C for 24 h. The mixture was then cooled to room temperature, diluted with water (1.50 L), and acidified with HCl (conc) to pH = 1. The mixture was then extracted with ethyl acetate (3 × 500 mL). The combined organic layers were washed with NaHCO_3_ (saturated aq, 500 mL) and water (500 mL), passed through a phase separator, concentrated, and dried under reduced pressure. The remaining dark brown solid was dissolved in toluene (200 mL) and passed through a short silica plug (5 cm × 10 cm in a glass frit filtration funnel) using toluene (3 × 100 mL) as eluent to afford dbCOT **2**. Yield: 11.2 g (80%). Isolated as a yellow semicrystalline solid, >95% pure by NMR spectroscopy and a single spot by TLC. ^1^H and ^13^C{^1^H} NMR spectroscopy data were in agreement with those previously reported.^45^ (5*E*,11*Z*)-dibenzo[*a*,*e*][8]annulene-5-carboxamide (**18**) was obtained by eluting the silica plug with methanol (2 × 100 mL). Yield: 1.29 g (7.6%). Isolated as a brown amorphous solid, >95% pure by NMR spectroscopy and a single spot by TLC. *R*_*f*_: 0.47 (95:5 CH_2_Cl_2_–MeOH). ^1^H NMR (CDCl_3_, 400 MHz): δ 8.01 (s, 1H), 7.27–7.19 (m, 2H), 7.19–7.14 (m, 4H), 7.14–7.11 (m, 1H), 7.09–7.04 (m, 1H), 6.84 (d, *J* = 11.8 Hz, 1H), 6.79–6.73 (m, 1H), 5.87 (br s, 1H), 5.31 (br s, 1H) ppm. ^13^C NMR (CDCl_3_, 101 MHz): δ 168.6, 140.7, 138.7, 137.1, 136.5, 136.2, 134.8, 133.8, 132.2, 129.5, 129.0, 128.9, 128.5, 128.2, 127.7, 127.4, 127.1 ppm. FTIR (CHCl_3_ film): 3463 (m), 3172 (br m), 3060 (w), 3018 (w), 1678 (s), 1631 (m), 1590 (m), 1367 (m) cm^–1^. HRMS (ESI-TOF) *m*/*z*: [M + H]^+^ calcd for C_17_H_14_NO 248.1075; found 248.1068.

### Dimethyl (5*E*,11*E*)-dibenzo[*a*,*e*][8]annulene-5,12-dicarboxylate (**17**)

An 87:13 mixture of hydrolysis products **1** and **16** (3.56 g) was dissolved in dry methanol (250 mL) at 0 °C, and thionyl chloride (2.60 mL, 35.6 mmol) was added dropwise over 10 min. The resulting solution was heated to reflux. After 3 h, TLC indicated complete conversion of the starting material. The reaction mixture was cooled to room temperature and NaHCO_3_ (aq saturated) was added to pH = 7. The volatiles were partially removed under reduced pressure. The resulting slurry was diluted with water (30 mL) and extracted with ethyl acetate (3 × 30 mL). The combined organic layers were passed through a phase separator, dry-loaded onto Celite, and purified using an automated flash purification system (25% ethyl acetate in heptane to 75% ethyl acetate in heptane) to afford dimethyl ester **17**. Yield: 2.10 g (67%). Isolated as a pale yellow amorphous solid, >95% pure by NMR spectroscopy and a single spot by TLC. *R*_*f*_: 0.64 (1:1 ethyl acetate–heptane). ^1^H NMR (CDCl_3_, 400 MHz): δ 7.98 (s, 2H), 7.26–7.20 (m, 4H), 7.18–7.09 (m, 4H), 3.80 (s, 6H) ppm. ^13^C{^1^H} NMR (CDCl_3_, 101 MHz): δ 167.2, 142.6, 135.5, 135.4, 134.9, 129.6, 128.2, 128.1, 127.3, 52.6 ppm. FTIR (CH_2_Cl_2_ film): 3058 (w), 3019 (w), 1716 (s), 1434 (m), 1255 (s), 1235 (m) cm^–1^. HRMS (ESI-TOF) *m*/*z*: [M + Na]^+^ calcd for C_20_H_16_O_4_Na 343.0946; found 343.0944.

### (5*E*,11*E*)-Dibenzo[*a*,*e*][8]annulene-5,12-dicarboxylic acid (**1**)

To a stirred solution of dimethyl ester **17** (2.09 g, 6.52 mmol) in methanol (20.0 mL) was added NaOH (26.0 mL, aq, 5.0 M). The resulting mixture was heated to reflux. After 4 h, TLC indicated full conversion of the starting material. The reaction mixture was cooled to room temperature, diluted with water (30 mL), and extracted with diethyl ether (3 × 30 mL) to remove any remaining starting material. The aqueous layer was poured over cold HCl (10 mL, aq, 1.0 M) and extracted with ethyl acetate (3 × 30 mL). The combined organic layers were passed through a phase separator and concentrated under reduced pressure to afford dicarboxylic acid **1**. Yield: 1.90 g (>95%). Isolated as a white amorphous solid, >95% pure by NMR spectroscopy and a single spot by TLC. *R*_*f*_: 0.24 (95:5 CH_2_Cl_2_–MeOH + 1% MeCO_2_H). ^1^H NMR (CD_3_OD, 400 MHz): δ 7.98 (s, 2H), 7.28–7.22 (m, 2H), 7.22–7.19 (m, 2H), 7.18–7.14 (m, 2H), 7.14–7.08 (m, 2H) ppm. ^13^C{^1^H} NMR (CD_3_OD, 101 MHz): δ 169.6, 143.3, 137.4, 136.8, 136.7, 130.6, 129.03, 128.99, 127.9 ppm. FTIR (neat): 3057 (w), 3018 (w), 2974 (br s), 1686 (s), 1636 (m), 1414 (w), 1279 (m), 1249 (m) cm^–1^. HRMS (ESI-TOF) *m*/*z*: [M – H]^−^ calcd for C_18_H_11_O_4_ 291.0657; found 291.0660.

### 5,10-Dihydroindeno[2,1-*a*]indene (**6**)

Dicarboxylic acid **1** (100 mg, 0.342 mmol) and Cu_2_O (3.70 mg, 25.8 μmol) were charged in a round-bottom flask, and dry *N*-methyl-2-pyrrolidone (7.50 mL) was added. The resulting red solution was heated to 190 °C for 24 h. The reaction mixture was then cooled down to room temperature, diluted with water (70 mL), acidified with HCl (conc) to pH = 1, and extracted with diethyl ether (3 × 50 mL). The combined organic layers were washed with NaHCO_3_ (saturated aq, 50 mL) and water (50 mL), passed through a phase separator, and concentrated under reduced pressure to afford indene **6**. Yield: 70.0 mg (>95%). Isolated as an off-white semicrystalline solid, >95% pure by NMR spectroscopy and a single spot by TLC. ^1^H and ^13^C{^1^H} NMR spectroscopy data were in agreement with those previously reported.^64^

### Dibenzo[*a*,*e*][8]annulene-5,11(6*H*,12*H*)-dione (**12**)

To a stirred solution of indene **6** (50.0 mg, 0.245 mmol) in a mixture of dichloromethane (1.20 mL), acetonitrile (1.20 mL), and water (1.80 mL) were added sodium periodate (220 mg, 1.03 mmol) and RuO_2_·*x*H_2_O (1.20 mg). The resulting mixture was stirred at room temperature for 4 h. The reaction mixture was then diluted with water (30 mL) and extracted with ethyl acetate (3 × 30 mL). The combined organic layers were washed with water (30 mL), passed through a phase separator, dry-loaded on Celite, and purified by an automated flash purification system (100% heptane to 8% ethyl acetate in heptane) to afford diketone **12**. Yield: 42.0 mg (73%). Isolated as an off-white semicrystalline solid, >95% pure by NMR spectroscopy and a single spot by TLC. ^1^H and ^13^C{^1^H} NMR spectroscopy data were in agreement with those previously reported.^34^

### (6*E*,14*E*)-Cycloocta[1,2-*b*:5,6-*b*′]dinaphthalene-6,15-dicarbonitrile (**21**)

To a stirred solution of dinitrile **19**(65) (106 mg, 0.514 mmol) and dialdehyde **20**(66,67) (99.4 mg, 0.540 mmol) in ethanol (60.0 mL) was added sodium ethoxide (21 wt % in ethanol, 96.0 μL, 0.257 mmol) dropwise over 1 min. The resulting orange mixture was stirred at room temperature for 3 h, then quenched with HCl (conc, 1.00 mL), and volatiles were partially removed under reduced pressure. The resulting slurry was partitioned between CH_2_Cl_2_ (30 mL) and water (30 mL). The aqueous layer was extracted with CH_2_Cl_2_ (3 × 30 mL). The combined organic layers were washed with water (30 mL) and brine (30 mL), passed through a phase separator, and concentrated under reduced pressure. The resulting brown solid was dry-loaded on Celite and purified using an automated flash purification system (100% heptane to 55% CH_2_Cl_2_ in heptane) to afford dinitrile **21**. Yield: 124 mg (68%). Isolated as an off-white amorphous solid, >95% pure by NMR spectroscopy and a single spot by TLC. *R*_*f*_: 0.26 (1:1 CH_2_Cl_2_–heptane). ^1^H NMR (CDCl_3_, 400 MHz): δ 7.93 (s, 2H), 7.91 (s, 2H), 7.82–7.77 (m, 2H), 7.77–7.72 (m, 2H), 7.68 (s, 2H), 7.54–7.44 (m, 4H) ppm. ^13^C{^1^H} NMR (CDCl3, 101 MHz): δ 147.2, 133.1, 132.8, 130.3, 129.5, 129.0, 128.6, 128.14, 128.06, 128.0, 119.3, 117.5 ppm. FTIR (CHCl_3_ film): 3055 (w), 3030 (w), 2215 (m), 1681 (s), 1524 (m), 1345 (m) cm^–1^. HRMS (ESI-TOF) *m*/*z*: [M + H]^+^ calcd for C_26_H_15_N_2_ 355.1235; found 355.1225.

### (6*Z*,14*Z*)-Cycloocta[1,2-*b*:5,6-*b*′]dinaphthalene (**5**)

Dinitrile **21** (53.8 mg, 0.152 mmol) was charged in a round-bottom flask, and glacial acetic acid (550 μL), water (550 μL), and concentrated sulfuric acid (550 μL) were added. The resulting solution was heated to reflux. After 30 h, the reaction was cooled to room temperature and poured onto ice (50 mL). The resulting mixture was extracted with ethyl acetate (3 × 50 mL). The combined organic layers were washed with brine (50 mL), passed through a phase separator, concentrated, and dried under reduced pressure to furnish a mixture of hydrolysis products as a brown solid (57.8 mg; ∼87% (6*E*,14*E*)-cycloocta[1,2-*b*:5,6-*b*′]dinaphthalene-6,15-dicarboxylic acid). This solid was charged in a round-bottom flask together with Cu_2_O (2.50 mg, 17.5 μmol) and 1,10-phenanthroline monohydrate (4.20 mg, 21.2 μmol), and *N*-methyl-2-pyrrolidone (7.50 mL) was added. The resulting red solution was heated to 190 °C for 24 h. The reaction was then cooled to room temperature, diluted with water (70 mL), acidified with HCl (conc) to pH = 1, and extracted with ethyl acetate (3 × 50 mL). The combined organic layers were washed with NaHCO_3_ (saturated aq, 50 mL) and water (50 mL), passed through a phase separator, and concentrated under reduced pressure. The resulting brown solid was dry-loaded on Celite and purified using an automated flash purification system (100% heptane to 2% CH_2_Cl_2_ in heptane) to afford dnCOT (**5**). Yield: 35.5 mg (77%). Isolated as a white crystalline solid, >95% pure by NMR spectroscopy and a single spot by TLC. ^1^H and ^13^C{^1^H} NMR spectroscopy data were in agreement with those previously reported.^17^

### (6*E*,12*E*)-Benzo[5,6]cycloocta[1,2-*b*]naphthalene-6,13-dicarbonitrile (**22**)

To a stirred solution of dialdehyde **14** (30.8 mg, 0.230 mmol) and dinitrile **19**(65) (43.0 mg, 0.208 mmol) in ethanol (50.0 mL) was added sodium ethoxide (21 wt % in ethanol, 39.0 μL, 0.104 mmol) dropwise over 1 min. After 3 h, the reaction was quenched with HCl (conc, 1.00 mL), and volatiles were partially removed under reduced pressure. The resulting slurry was partitioned between CH_2_Cl_2_ (30 mL) and water (30 mL). The aqueous layer was extracted with CH_2_Cl_2_ (3 × 30 mL). The combined organic layers were washed with water (30 mL) and brine (30 mL), passed through a phase separator, and concentrated under reduced pressure. The resulting brown solid was dry-loaded on Celite and purified using an automated flash purification system (100% heptane to 45% CH_2_Cl_2_ in heptane) to afford dinitrile **22**. Yield: 37.9 mg (60%). Isolated as an off-white amorphous solid, >95% pure by NMR spectroscopy and a single spot by TLC. *R*_*f*_: 0.40 (1:1 CH_2_Cl_2_–heptane). ^1^H NMR (CDCl_3_, 400 MHz): δ 7.91 (s, 2H), 7.86–7.78 (m, 2H), 7.69 (s, 2H), 7.58–7.50 (m, 2H), 7.35–7.28 (m, 2H), 7.20–7.13 (m, 2H) ppm. ^13^C{^1^H} NMR (CDCl_3_, 101 MHz): δ 147.2, 133.3, 133.1, 129.7, 129.4, 129.2, 128.7, 128.2, 128.1, 119.1, 117.4 ppm. FTIR (CHCl_3_ film): 3056 (w), 3026 (w), 2218 (m), 1632 (w), 1620 (w), 1489 (w) cm^–1^. HRMS (ESI-TOF) *m*/*z*: [M + H]^+^ calcd for C_22_H_13_N_2_ 305.1079; found 305.1073.

### (6*Z*,12*Z*)-benzo[5,6]cycloocta[1,2-*b*]naphthalene (**23**)

Dinitrile **22** (57.3 mg, 0.188 mmol) was charged in a round-bottom flask, and glacial acetic acid (700 μL), water (700 μL), and concentrated sulfuric acid (700 μL) were added. The resulting mixture was heated to reflux for 30 h, then cooled to room temperature, and poured onto ice (50 mL). The mixture was extracted with ethyl acetate (3 × 50 mL). The combined organic layers were washed with brine (50 mL), passed through a phase separator, concentrated, and dried under reduced pressure to furnish a mixture of hydrolysis products as a brown solid (62.0 mg; ∼87% (6*E*,12*E*)-benzo[5,6]cycloocta[1,2-*b*]naphthalene-6,13-dicarboxylic acid). This solid was charged in a round-bottom flask together with Cu_2_O (3.20 mg, 22.4 μmol) and 1,10-phenanthroline monohydrate (5.40 mg, 27.2 μmol), and *N*-methyl-2-pyrrolidone (10.0 mL) was added. The resulting red solution was heated to 190 °C for 24 h. The reaction mixture was then cooled to room temperature, diluted with water (100 mL), acidified with HCl (conc) to pH = 1, and extracted with ethyl acetate (3 × 50 mL). The combined organic layers were washed with NaHCO_3_ (saturated aq, 50 mL) and water (50 mL), passed through a phase separator, and concentrated under reduced pressure. The resulting brown solid was dry-loaded on Celite and purified using an automated flash purification system (100% heptane to 2% CH_2_Cl_2_ in heptane) to afford bnCOT (**23**). Yield: 35.4 mg (74%). Isolated as an off-white semicrystalline solid, >95% pure by NMR spectroscopy and a single spot by TLC. *R*_*f*_: 0.37 (heptane). ^1^H NMR (CDCl_3_, 400 MHz): δ 7.74–7.65 (m, 2H), 7.56 (s, 2H), 7.41–7.32 (m, 2H), 7.18–7.06 (m, 4H), 6.98 (d, *J* = 12.0 Hz, 2H), 6.86 (d, *J* = 12.0 Hz, 2H) ppm. ^13^C{^1^H} NMR (CDCl_3_, 101 MHz): δ 136.8, 135.3, 133.7, 132.9, 132.4, 129.1, 128.1, 127.6, 127.0, 126.1 ppm. FTIR (CHCl_3_ film): 3053 (w), 3009 (w), 1491 (w), 1274 (w) cm^–1^. HRMS (ESI-TOF) *m*/*z*: [M + H]^+^ calcd for C_20_H_15_ 255.1174; found 255.1173. mp: 151–152 °C (recrystallized from ethanol).

## Supporting Information Available

Alternative synthetic procedures for dbCOT (**2**), extended optimization of the decarboxylation of **1**, optimization of the hydrolysis of dinitrile **15**, crystallographic information for **12**, and copies of ^1^H and ^13^C{^1^H} NMR spectra (PDF)

## Accession Codes

CCDC 2145274 contains the supplementary crystallographic data for this paper. These data can be obtained free of charge via www.ccdc.cam.ac.uk/data_request/cif, or by emailing data_request@ccdc.cam.ac.uk, or by contacting The Cambridge Crystallographic Data Centre, 12 Union Road, Cambridge CB2 1EZ, UK; fax: +44 1223 336033.
