# Supplementary material for: Copper(I) Catalyzed Decarboxylative Synthesis of Diareno[a,e]cyclooctatetraenes
Source: J Org Chem. 2022 May 19;87(11):7501–8. doi: 10.1021/acs.joc.2c00286 (PMC9490866; doi:10.1021/acs.joc.2c00286)
Supplement: Supplementary file 1 — jo2c00286_si_001.pdf [file jo2c00286_si_001.pdf]

Supporting Information

Copper(I) Catalyzed Decarboxylative Synthesis of Diareno[*a*,*e*]cyclooctatetraenes

Magdalena Tasić, Albert Ruiz-Soriano, and Daniel Strand*

Centre for Analysis and Synthesis, Department of Chemistry, Lund University, Box 124, SE-221 00 Lund, Sweden.

E-mail: Daniel.Strand@chem.lu.se

Table of Contents:

I.	*General methods*	*S2*
II.	*Alternative synthetic procedures to form dbCOT (2)*	*S3*
III.	*Optimization of the decarboxylation of **1**, contd.*	*S5*
IV.	*Optimization of the hydrolysis of dinitrile **15***	*S5*
V.	*Single crystal X-ray diffraction (scXRD) analysis of diketone **12***	*S7*
VI.	*^1H and $^{13}C\{^1H\}$ NMR spectra for **1-2**, **5-6**, **12**, **15**, **17-18**, and **21-23***	*S9*

SAFETY STATEMENT: No unusual or unexpected safety issues were encountered in this work.

I. General methods

Unless otherwise noted, air- and moisture-sensitive reactions were carried out in oven-dried (>110 °C) glassware under a positive pressure of nitrogen. Air- and moisture-sensitive reagents, solvents, and solutions were transferred *via* syringe or stainless-steel cannula under a dry nitrogen atmosphere. Unless otherwise noted, reactions with a total volume above 5 mL were conducted in round bottom flasks equipped with a magnetic stir bar. Reactions with a total volume below 5 mL were conducted in Biotage® microwave vessels equipped with a magnetic stir bar and sealed with a septum crimp cap. Reactions were stirred using oven-dried Teflon®-coated magnetic stir bars. Room temperature indicates a temperature in the span 20 – 25 °C. Elevated temperatures were maintained using thermostat-controlled heating plate and aluminum heating blocks. Commercial reagents and solvents were used as received without further purification unless otherwise noted. MeOH was obtained from an Inert PureSolv Micro solvent purification system. Analytical thin-layer chromatography (TLC) was performed on

silica-backed TLC plates (60 F_{254} silica gel) and visualized by UV-light (λ = 254 nm) and/or treatment with either *p*-anisaldehyde stain, $KMnO_4$ stain, or phosphomolybdic acid (PMA) stain followed by gentle heating. Preparative column chromatography was performed using a Biotage® Isolera™ One system. NMR spectra were recorded on a 400 MHz (^{1}H at 400 MHz and ^{13}C at 101 MHz) Bruker Avance II spectrometer at *T* = 298 K. Spectra were processed using Mnova v14.1. ^{1}H NMR chemical shifts are referenced to the residual solvent peak as internal standard ($CDCl_3$ = 7.26 ppm; CD_3OD = 3.31 ppm) and are listed as follows: chemical shifts (δ, ppm), multiplicity, scalar coupling constant(s) in Hz, and integral values. ^{13}C NMR (^{1}H decoupled) chemical shifts are referenced to the residual solvent peak as internal standard ($CDCl_3$ = 77.16 ppm; CD_3OD = 49.0 ppm). FTIR spectra were recorded on a Bruker Alpha II spectrometer as thin films using the ATR attachment and are reported as follows: wavenumbers (cm^{-1}), description (w = weak, m = medium, s = strong, br = broad). Melting points were measured on a BÜCHI B-540 melting point apparatus and are uncorrected. High-resolution mass spectra (HRMS) were recorded on an ESI-QTOF mass spectrometer (Waters Xevo-G2), in positive or negative mode between m/z 50-1200, employing lockmass correction according to the manufacturer's instructions.

*II. Alternative synthetic procedures to form dbCOT (**2**)*

(5*Z*,11*Z*)-Dibenzo[*a*,*e*][8]annulene (dbCOT, 2). *Gram scale procedure for decarboxylation of purified dicarboxylic acid **1**.* The dicarboxylic acid **1** (3.02 g, 10.3 mmol) was charged in a round bottom flask together with Cu_2O (112 mg, 0.783 mmol) and 1,10-phenanthroline monohydrate (306 mg, 1.54 mmol), and dry *N*-methyl-2-pyrrolidone (225 ml) was added. The resulting red solution was stirred at 190 °C for 16 h. The reaction mixture was then cooled down to room temperature, diluted with water (200 mL), acidified with HCl (conc.) until pH = 1, and extracted with diethyl ether (3 x 100 mL). The combined organic layers were washed with

$NaHCO_3$ (sat. aq., 50 mL) and water (50 mL), passed through a phase separator, and concentrated under reduced pressure to afford dbCOT (**2**). **Yield:** 2.04 g (97%). Isolated as a yellow semi-crystalline solid, >95% pure by NMR and single spot by TLC.

*Procedure for conversion of carboxamide **18** to dbCOT (2).* To a stirred solution of carboxamide **18** (1.29 g, 5.22 mmol) in methanol (15.5 mL) was added NaOH (36.0 mL, aq., 5.80 M). The resulting brown mixture was heated to reflux for 24 h. The reaction mixture was then cooled to room temperature and extracted with ethyl acetate (50 mL) to remove any remaining starting material. The aqueous layer was diluted with water (150 mL) and acidified with HCl (conc.) to pH = 1. The mixture was then extracted with ethyl acetate (3 x 50 mL). The combined organic layers were washed with brine (50 mL), passed through a phase separator, concentrated, and dried under reduced pressure to obtain a brown solid (880 mg). This solid was charged in a round bottom flask together with Cu_2O (63.4 mg, 0.443 mmol) and 1,10-phenanthroline monohydrate (105 mg, 0.530 mmol), and *N*-methyl-2-pyrrolidone (26.0 ml) was added. The resulting red solution was heated to 190 °C for 24 h. The reaction mixture was then cooled to room temperature, diluted with water (75.0 mL), acidified with HCl (conc.) to pH = 1, and extracted with diethyl ether (2 x 50 mL) and ethyl acetate (1 x 50 mL). The combined organic layers were washed with $NaHCO_3$ (sat. aq., 50 mL) and water (50 mL), passed through a phase separator, and concentrated under reduced pressure. The resulting brown solid was dissolved in toluene (50 mL) and passed through a short silica plug (5 x 5 cm in a glass frit filter funnel) eluting with toluene (3 x 50 mL) to afford dbCOT (**2**). **Yield:** 0.48 g (45%). Isolated as an orange semi-crystalline solid, >95% pure by NMR and single spot by TLC.

^{1}H and $^{13}C\{^{1}H\}$ NMR data were in agreement with those previously reported.[1]

---

[1] Franck, G.; Brill, M.; Helmchen, G. Dibenzo[*a*,*e*]cyclooctene: Multi-gram Synthesis of a Bidentate Ligand. *Org. Synth.* **2012**, *89*, 55-65.

III. *Optimization of the decarboxylation of **1**, contd.*

Table S1. Optimization of the decarboxylation of dicarboxylic acid **1**, contd.

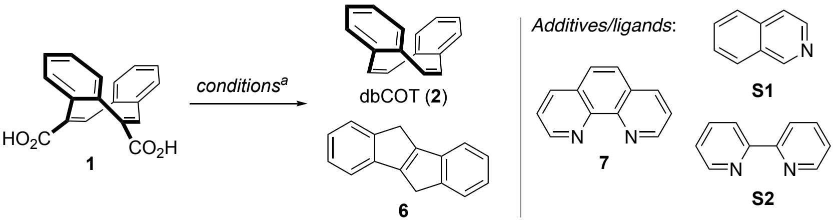

Entry	Catalyst (mol%)	Additives (mol%)	Solvent	Temp. (°C)	Selectivity **2**:**6**	Product, yield[b] (%)
1[c]	$Cu(OH)_2$ (10)	**7** (10), **S1** (100)	NMP	150	100:0	**2**, 2
2[c]	$Cu(OH)_2$ (10)	**7** (10), K_2CO_3 (100)	NMP	150	-	-
3[c]	$Cu(OH)_2$ (10)	**7** (10)	NMP	150	7:93	**6**, 4
4[c]	$Cu(OH)_2$ (10)	**S2** (30)	NMP	150	-	-
5[d]	Cu_2O (5)	**7** (10), **S1** (430)	NMP	170	25:75	**2**, 23
6[d]	AgOAc (10)	K_2CO_3 (15)	NMP	120	-	-
7[e]	Ag_2CO_3 (10)	-	DMSO	120	-	-
8[f]	$Pd(TFA)_2$ (20)	TFA (10)	DMSO/DMF	70	-	-

(a) *General conditions:* Dicarboxylic acid **1** (4.6 mM), catalyst, additives and solvent, were stirred at the indicated temperature under an inert atmosphere for 24 h. (b) Yield of the major product as determined by ^{1}H NMR analysis using 1-methoxynaphthalene as an internal standard. (c) For conditions, see reference 2. (d) For conditions, see reference 3. (e) For conditions, see reference 4. (f) For conditions, see reference 5.

IV. *Optimization of the hydrolysis of dinitrile **15***

General procedure: To dinitrile **15** was added a mixture of reagents and solvents to the concentration indicated. The reaction mixture was then heated. After the indicated time, the

---

[2] Cadot, S.; Rameau, N.; Mangematin, S.; Pinel, C.; Djakovitch, L. Preparation of functional styrenes from biosourced carboxylic acids by copper catalyzed decarboxylation in PEG. *Green Chem.* **2014**, *16*, 3089-3097.
[3] Gooßen, L. J.; Rodríguez, N.; Linder, C.; Lange, P. P.; Fromm, A. Comparative Study of Copper- and Silver-Catalyzed Protodecarboxylations of Carboxylic Acids. *ChemCatChem* **2010**, *2*, 430-442.
[4] Cornella, J.; Sanchez, C.; Banawa, D.; Larrosa, I. Silver-catalysed protodecarboxylation of ortho-substituted benzoic acids. *Chem. Commun.* **2009**, 7176-7178.
[5] Dickstein, J. S.; Mulrooney, C. A.; O'Brien, E. M.; Morgan, B. J.; Kozlowski, M. C. Development of a Catalytic Aromatic Decarboxylation Reaction. *Org. Lett.* **2007**, *9*, 2441-2444.

mixture was cooled to room temperature and poured onto ice. The resulting precipitate was collected by filtration. The residue was dissolved in ethyl acetate and extracted with $NaHCO_3$ (sat. aq.). The aqueous layer was then acidified with HCl (conc.) to pH = 1 and extracted three times with ethyl acetate. The combined organic layers were passed through a phase separator, evaporated, and dried.

Table S2. Optimization of the hydrolysis conditions of **15**

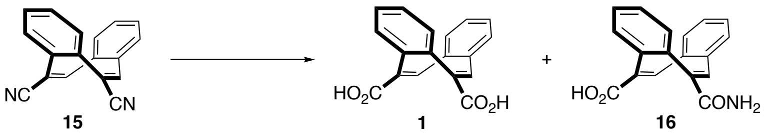

Entry	Reagent (mL)	Solvents (mL)	**15** (mmol)	Time (h)	Temp. (°C)	**1**:**16**[a]	Isolated yield for the mixture of **1** and **16** (%)
1	H_2SO_4 (26)	H_2O (26)/$MeCO_2H$ (26)	14	30	reflux	95:5[b]	92[c]
2[d]	H_2SO_4 (7.6)	H_2O (7.6)/$MeCO_2H$ (7.6)	3.9	22	120	92:8[b]	84
3	H_2SO_4 (80)	H_2O (75)	3.9	22	120	89:11[b]	78
4	H_2SO_4 (80)	H_2O (75)	3.9	5	120	77:23[b]	66
5	H_2SO_4 (8.0)	H_2O (7.5)	0.39	5	100	34:66	59
6	H_2SO_4 (8.0)	H_2O (7.5)	0.39	5	120	87:13[b]	78
7[e]	H_2SO_4 (8.0)	H_2O (7.5)	0.39	5	135	87:13[b]	75
8	H_2SO_4 (4.0)	H_2O (12)	0.39	5	135	100:0	14
9	H_2SO_4 (20)	-	0.39	5	135	-[b]	-
10[f]	HCl conc. (3.4)	H_2O (0.6)	3.9	19	50	-	-
11	HCl conc. (4.4)	$MeCO_2H$ (1.0)	3.9	19	50	4:96	2.8
12[g]	HCl conc. (23)	-	0.39	28	100	100:0	traces
13[h]	H_3PO_4 (8 equiv.)	-	0.39	5	155	34:66[b]	62
14[h]	H_3PO_4 (25 equiv.)	-	0.39	24	175	75:25[b]	70
15	H_3PO_4 (8 equiv.)	$MeCO_2H$ (0.20)	0.39	5	120	-	-
16[i]	NaOH (11 equiv.)	H_2O (3.4)	0.39	5	100	-[b]	-
17[j]	KOH (40 equiv.)	H_2O (2.5)/diethylene glycol (12.5)	0.39	24	135	100:0[b]	26

(a) Ratio determined by integration of the corresponding peaks in the crude ^1H NMR spectrum of the precipitate collected upon pouring onto ice. (b) Complete consumption of **15** observed. (c) After pouring onto ice, the filtrate was extracted with diethyl ether, concentrated, and the resulting solid residue combined with the precipitate. (d)

For conditions, see reference 6. (e) For conditions, see reference 7. (f) For conditions, see reference 8. (g) For conditions, see reference 9. (h) For conditions, see reference 10. (i) For conditions, see reference 11. (j) For conditions, see reference 12.

*V. Single crystal X-ray diffraction (scXRD) analysis of diketone **12***

Single crystals covered in paratone oil were cut to size and mounted on a MiTeGen micro-mount loop. Data collection was performed on an Agilent Xcalibur Sapphire3 equipped with a MoKα high-brilliance IµS radiation source (λ = 0.71073 Å). Absorption was corrected for using multi-scan empirical absorption correction with spherical harmonics as implemented in the SCALE3 ABSPACK scaling algorithm.[13] The structures were solved in WinGX[14] using SUPERFLIP[15] or SHELXL[16] 2016/4 and refined using SHELXL 2016/4. Non-hydrogen atoms were refined anisotropically.

---

6 Ismail, M. A. H.; Barker, S.; Abou El Ella, D. A.; Abouzid, K. A. M.; Toubar, R. A.; Todd, M. H. Design and Synthesis of New Tetrazolyl- and Carboxy-biphenylylmethyl-quinazolin-4-one Derivatives as Angiotensin II AT1 Receptor Antagonists. *J. Med. Chem.* **2006**, *49*, 1526-1535.

7 Fieser, L. F.; Pechet, M. M. 1,2,5,6-Dibenzcycloöctatetraene. *J. Am. Chem. Soc.* **1946**, *68*, 2577-2580.

8 Krieble, V. K.; Noll, C. I. The Hydrolysis of Nitriles with Acids. *J. Am. Chem. Soc.* **1939**, *61*, 560-563.

9 Newkome, G. R.; Moorefield, C. N.; Theriot, K. J. A convenient synthesis of bis-homotris: 4-amino-4-[1-(3-hydroxypropyl)]-1,7-heptanediol, and 1-azoniapropellane. *J. Org. Chem.* **1988**, *53*, 5552-5554.

10 Berger, G.; Olivier, S. C. J. Une Nouvelle Méthode de Saponification des Amides et des Nitriles. *Recl. Trav. Chim. Pays-Bas* **1927**, *46*, 600-604.

11 Akira, K.; Hasegawa, H.; Baba, S. Synthesis of selectively 13C-labelled benzoic acid for nuclear magnetic resonance spectroscopic measurement of glycine conjugation activity. *J. Labelled Compd. Radiopharm.* **1995**, *36*, 845-853.

12 Buckland, P. R.; Hacker, N. P.; McOmie, J. F. W. Biphenylenes. Part 32. A new, general synthesis of mono- and poly-benzobiphenylenes from substituted benzocyclobutene-1,2-diones and ortho-bis(cyanomethyl)arenes. *J. Chem. Soc., Perkin Trans. 1* **1983**, 1443-1448.

13 CrysAlis PRO. Agilent Technologies **2011**.

14 Farrugia, L. WinGX and ORTEP for windows: an update. *J. Appl. Crystallogr.* **2012**, *45*, 849-854.

15 Palatinus, L.; Chapuis, G. SUPERFLIP - A computer program for the solution of crystal structures by charge flipping in arbitrary dimensions. *J. Appl. Crystallogr.* **2007**, 40, 786-790.

16 Sheldrick, G. A Short History of ShelX. *Acta Crystallogr. A* **2008**, *64*, 112-122.

Table S3. Crystal data for diketone **12**. *Crystallization conditions:* Single crystals suitable for scXRD analysis were grown at room temperature from a slowly evaporating mixture of hexane and CH_2Cl_2.

Chemical formula	$C_{16}H_{12}O_2$	
Formula weight	236.26	
Collection temperature /K	293(2)	
Crystal size /mm^3	0.2 x 0.2 x 0.1	
Crystal habit	yellow, block	
Wavelength /Å	0.71073	
Crystal system	Monoclinic	
Space group	*C*2/*c*	
Unit cell dimensions:	a = 18.7567(13) Å	α = 90 °
	b = 4.9242(3) Å	β = 116.459(9) °
	c = 14.2919(10) Å	γ = 90 °
Unit cell volume /Å^3	1181.76(16)	
Z, Calculated density /Mg/m^3	4, 1.328	
Radiation type	MoKα	
Absorption coefficient, m/mm^{-1}	0.087	
No. reflections collected / unique	4970/ 1417	
R_{int}	0.0345	
Completeness to theta = 25.000 /%	99.9	
Data / restraints / parameters	1417/ 0 / 83	
Goodness of fit on F^2	1.028	
Final R indices $(I > 2\sigma(I))$	R_1 = 0.0512, wR_2 = 0.1086	
R indices *(all data)*	R_1 = 0.0847, wR_2 = 0.1290	
Largest diff. peak and hole /e-/Å^3	0.154 and -0.141	
CCDC	2145274	

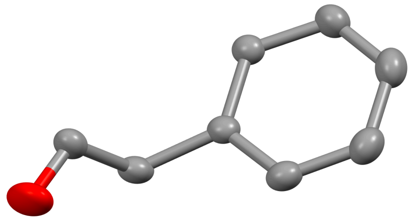

Figure S1. Asymmetric unit of **12**. Black = carbon atom; red = oxygen atom. Thermal ellipsoids are shown at 30% probability. Hydrogen atoms are omitted for clarity.

*VI. ^{1}H and $^{13}C\{1H\}$ NMR spectra for **1-2**, **5-6**, **12**, **15**, **17-18**, and **21-23***

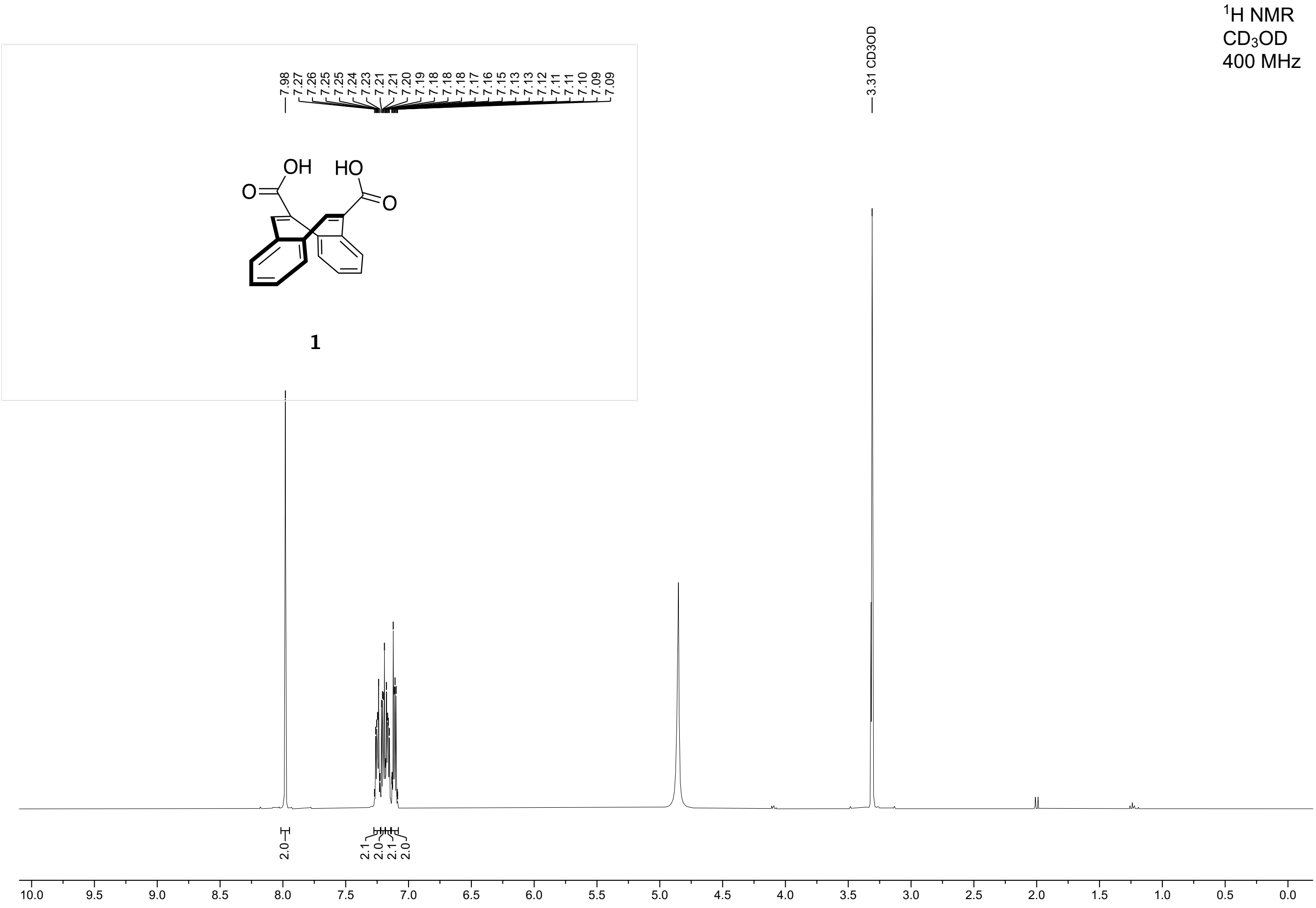
^{1}H NMR
CD$_3$OD
400 MHz
7.98
7.27 7.26 7.25 7.25 7.24 7.23 7.21 7.21 7.20 7.19 7.18 7.18 7.18 7.17 7.16 7.15 7.13 7.13 7.12 7.11 7.11 7.10 7.09 7.09
3.31 CD3OD
OH
HO
O
O
1
2.0
2.1 2.0 2.1 2.0

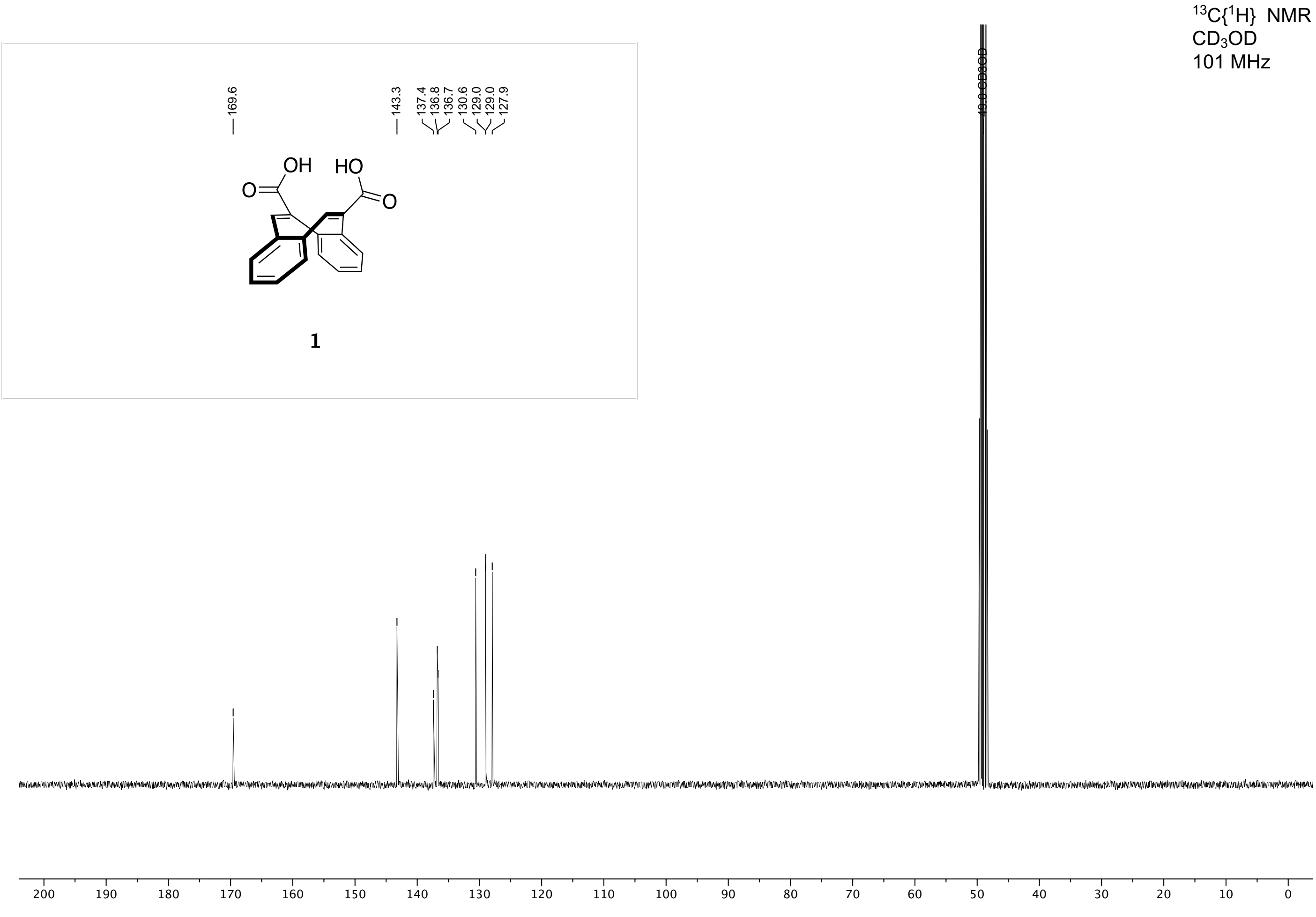
^{13}C{^{1}H} NMR
CD$_3$OD
101 MHz
169.6
143.3
137.4 136.8 136.7 130.6 129.0 129.0 127.9
49.0 CD3OD
OH
HO
O
O
1

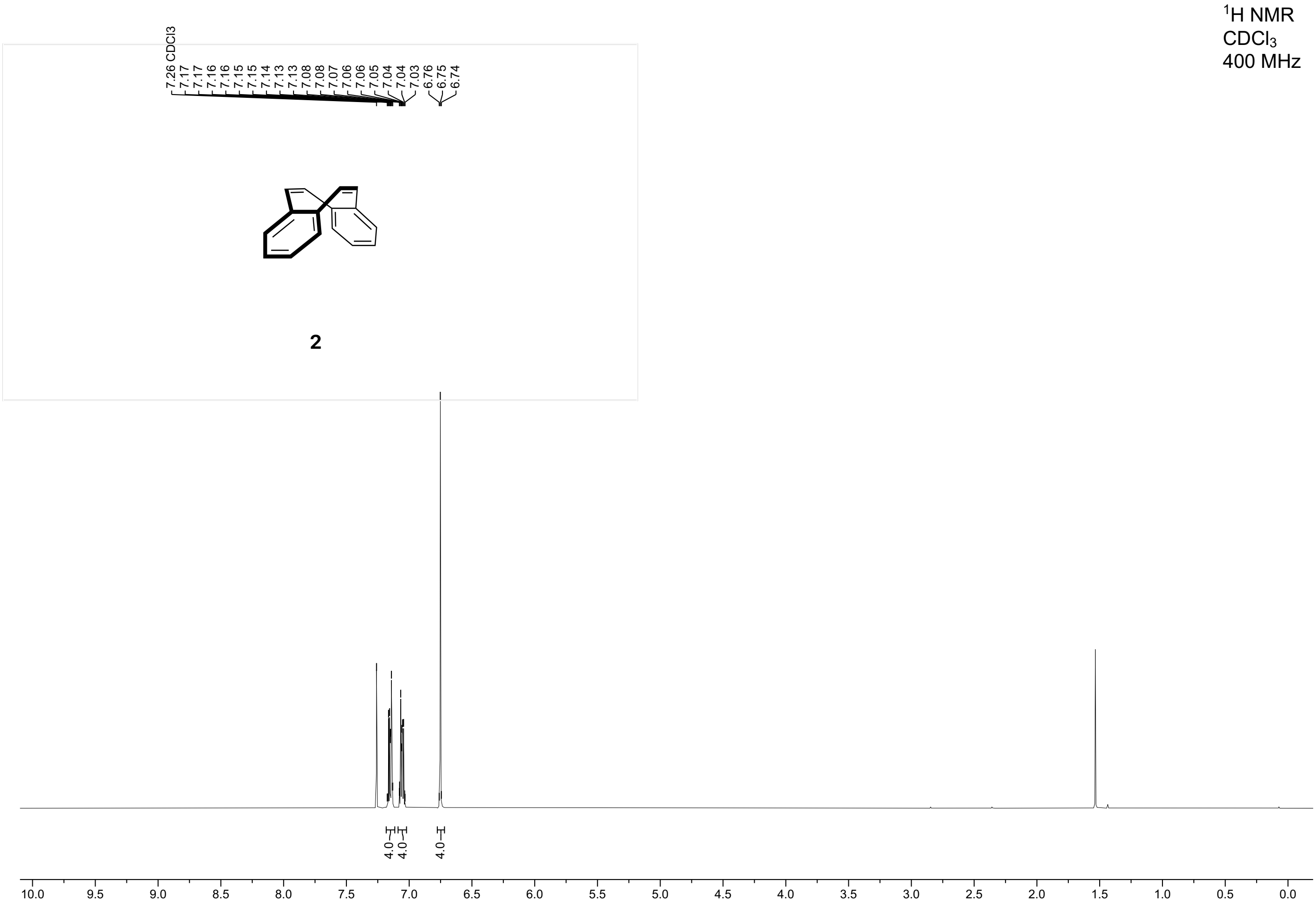

^{1}H NMR
$CDCl_3$
400 MHz
7.26 CDCl3
7.17
7.17
7.16
7.15
7.15
7.14
7.13
7.13
7.08
7.08
7.07
7.06
7.06
7.05
7.04
7.04
7.03
6.76
6.75
6.74
2
4.0
4.0
4.0
10.0
9.5
9.0
8.5
8.0
7.5
7.0
6.5
6.0
5.5
5.0
4.5
4.0
3.5
3.0
2.5
2.0
1.5
1.0
0.5
0.0

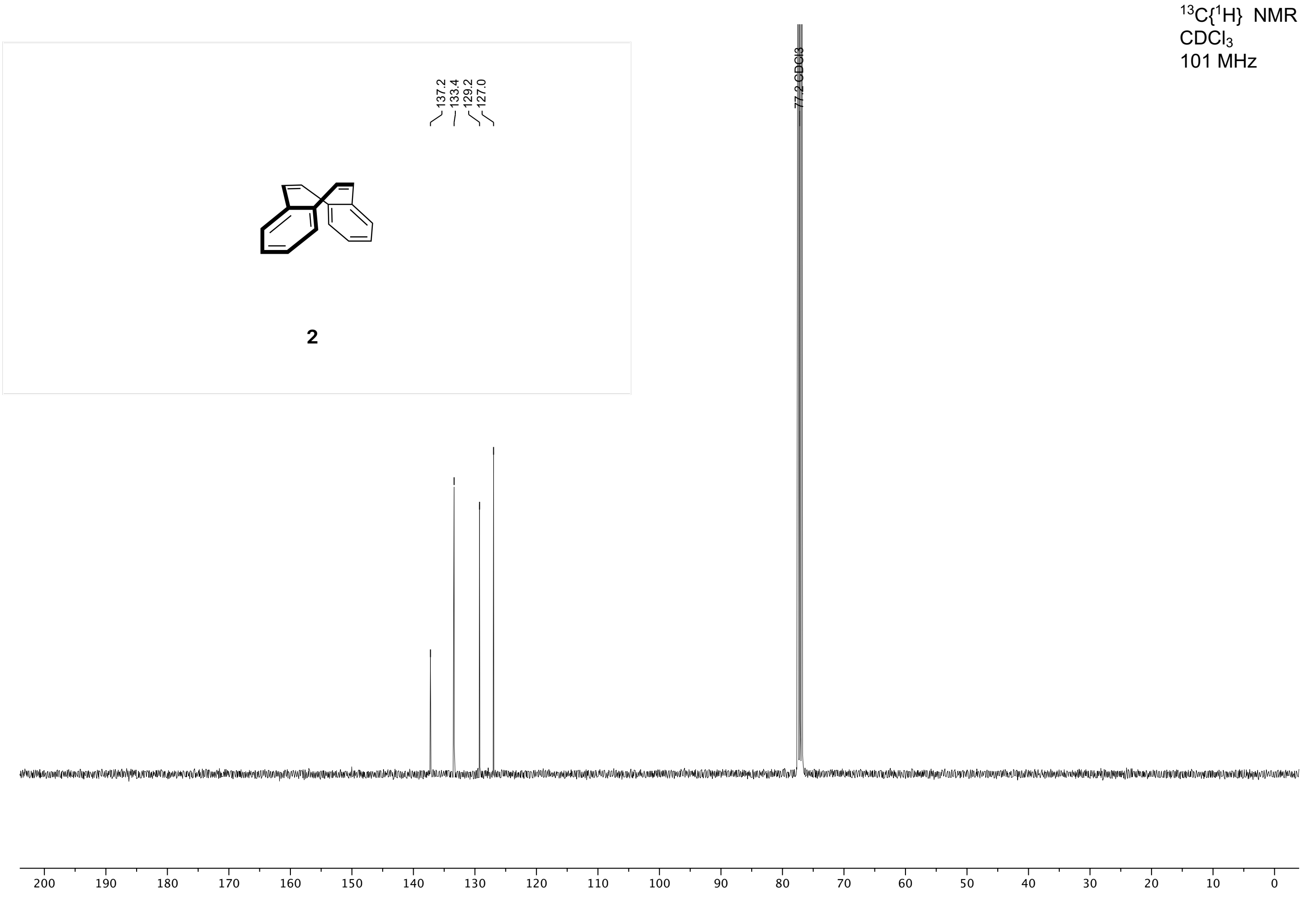

$^{13}C\{^1H\}$ NMR
$CDCl_3$
101 MHz
77.2 CDCl3
137.2
133.4
129.2
127.0
2
200
190
180
170
160
150
140
130
120
110
100
90
80
70
60
50
40
30
20
10
0

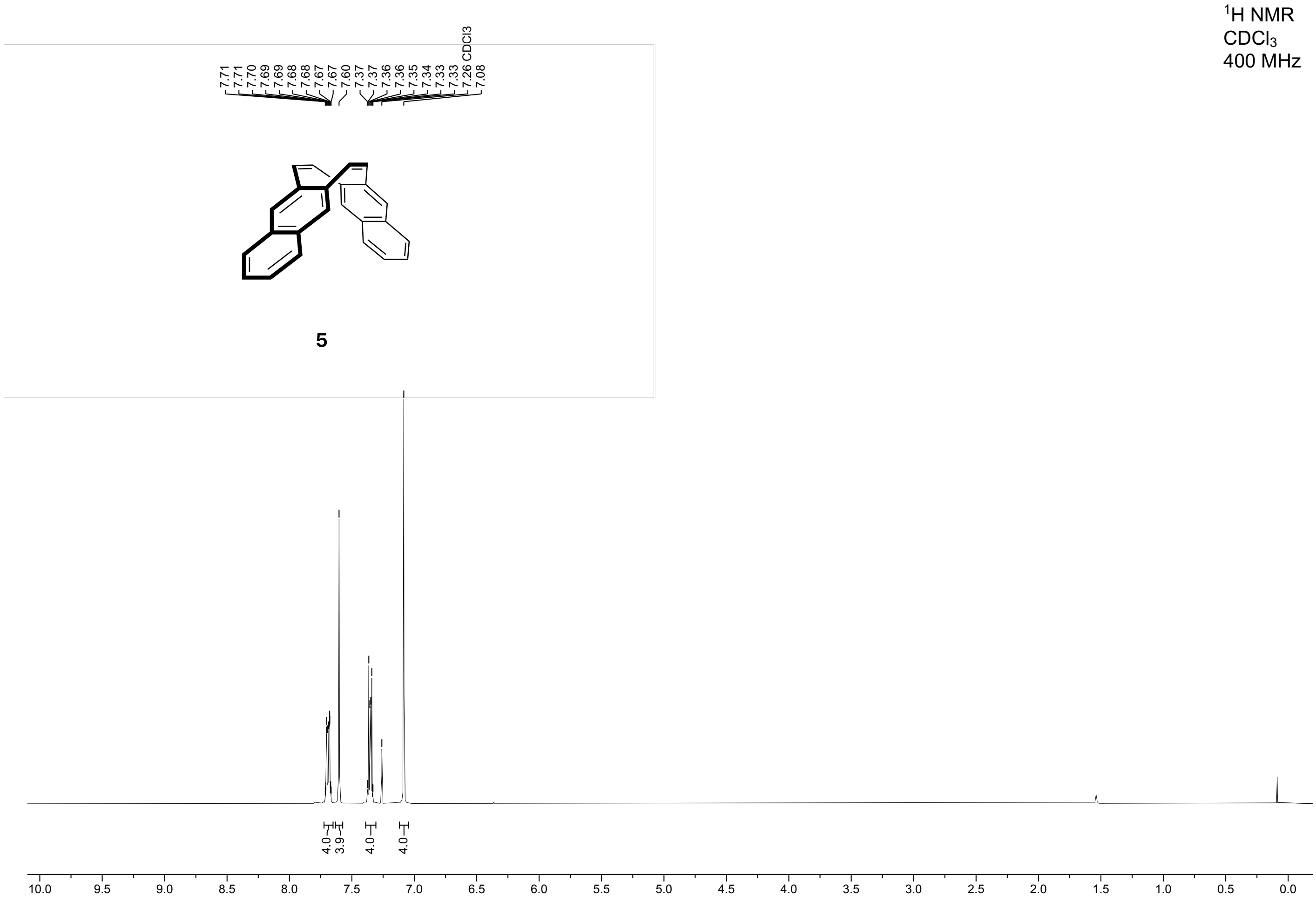
^{1}H NMR
$CDCl_3$
400 MHz
7.71 7.71 7.70 7.69 7.69 7.68 7.68 7.67 7.67 7.60 7.37 7.37 7.36 7.36 7.35 7.34 7.33 7.33 7.26 CDCl3 7.08
5
4.0 3.9 4.0 4.0
10.0 9.5 9.0 8.5 8.0 7.5 7.0 6.5 6.0 5.5 5.0 4.5 4.0 3.5 3.0 2.5 2.0 1.5 1.0 0.5 0.0

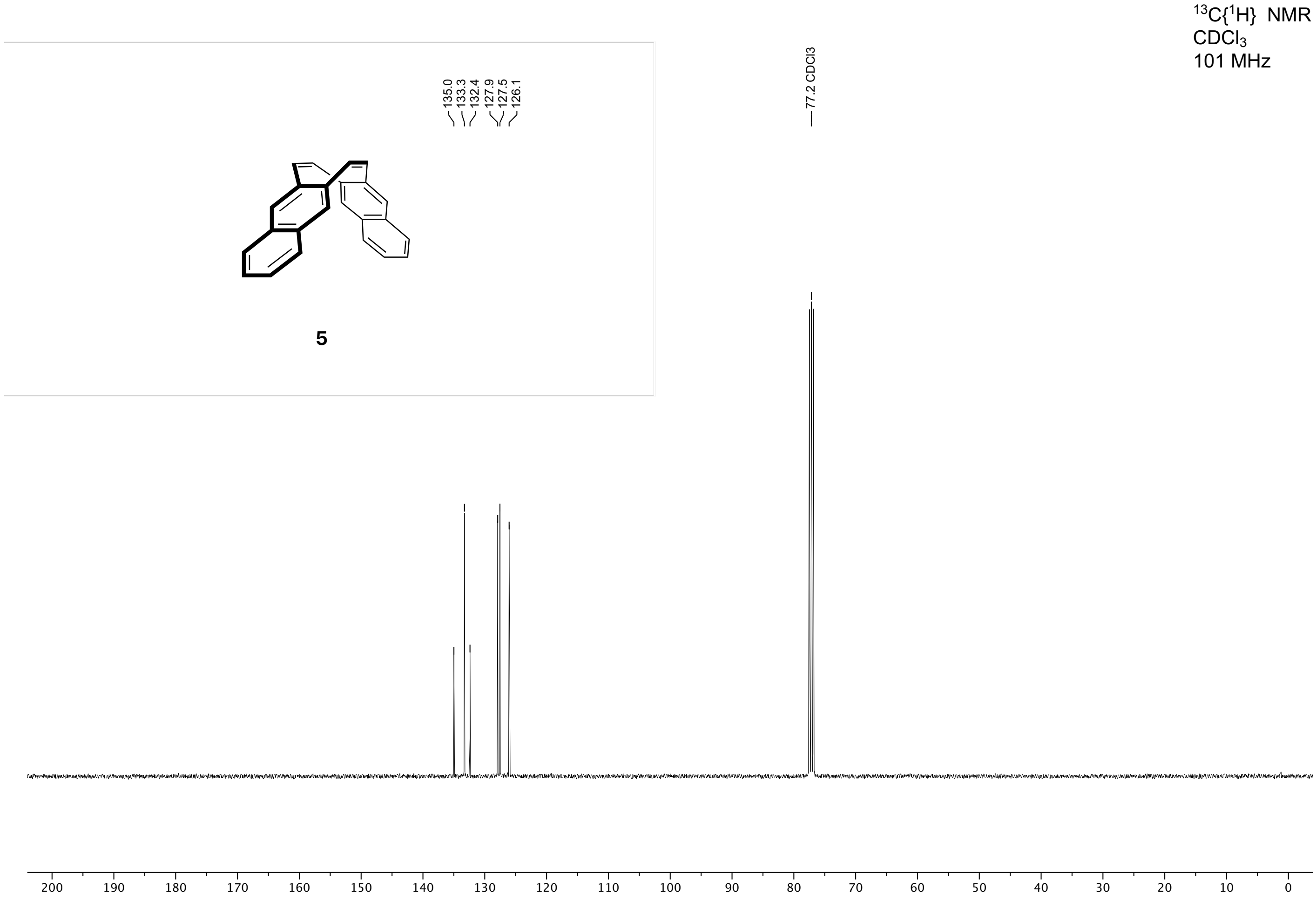
^{13}C{^{1}H} NMR
$CDCl_3$
101 MHz
135.0 133.3 132.4 127.9 127.5 126.1
77.2 CDCl3
5
200 190 180 170 160 150 140 130 120 110 100 90 80 70 60 50 40 30 20 10 0

^{1}H NMR
$CDCl_3$
400 MHz

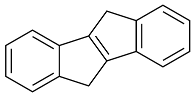

6

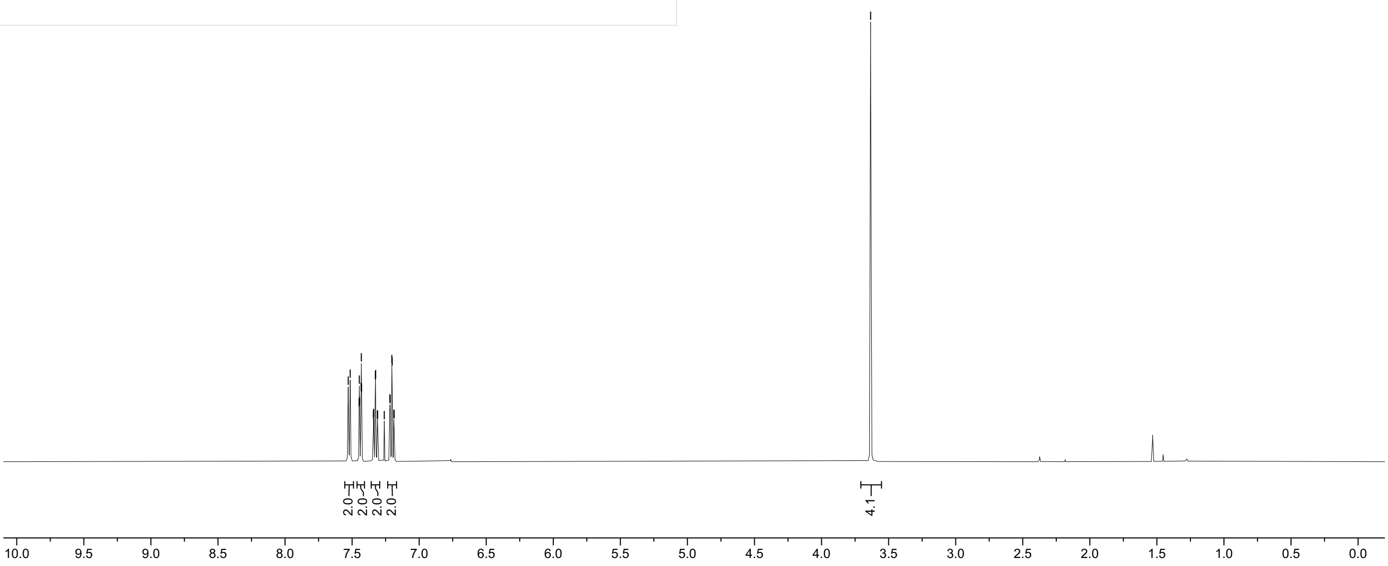

$^{13}C\{^{1}H\}$ NMR
$CDCl_3$
101 MHz

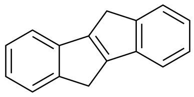

6

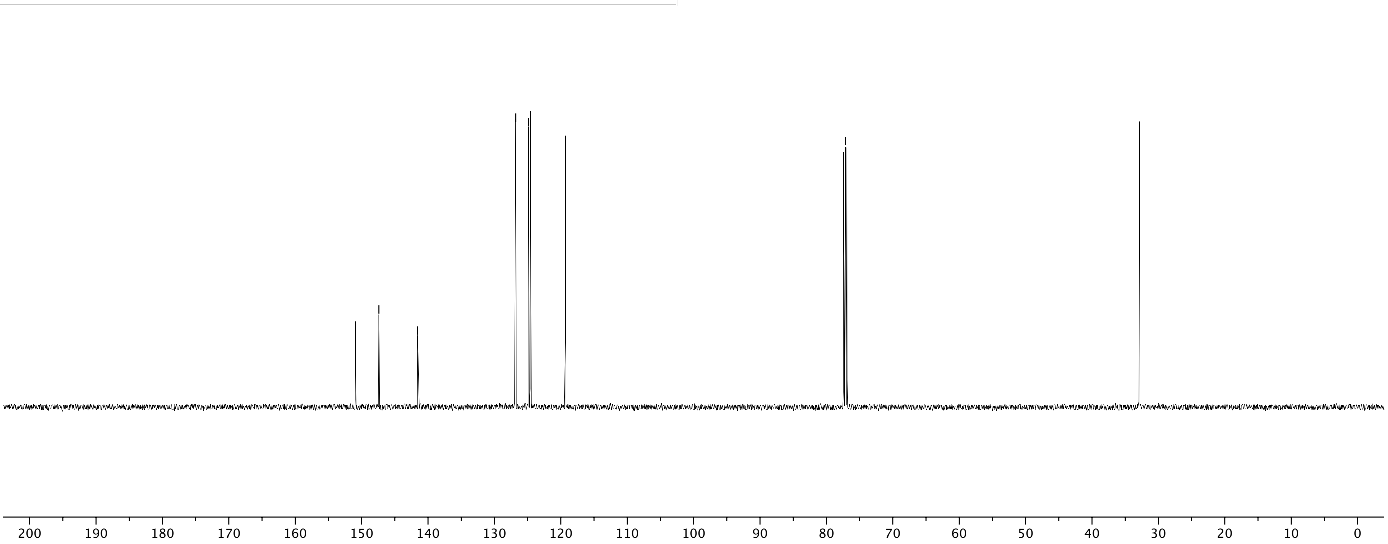

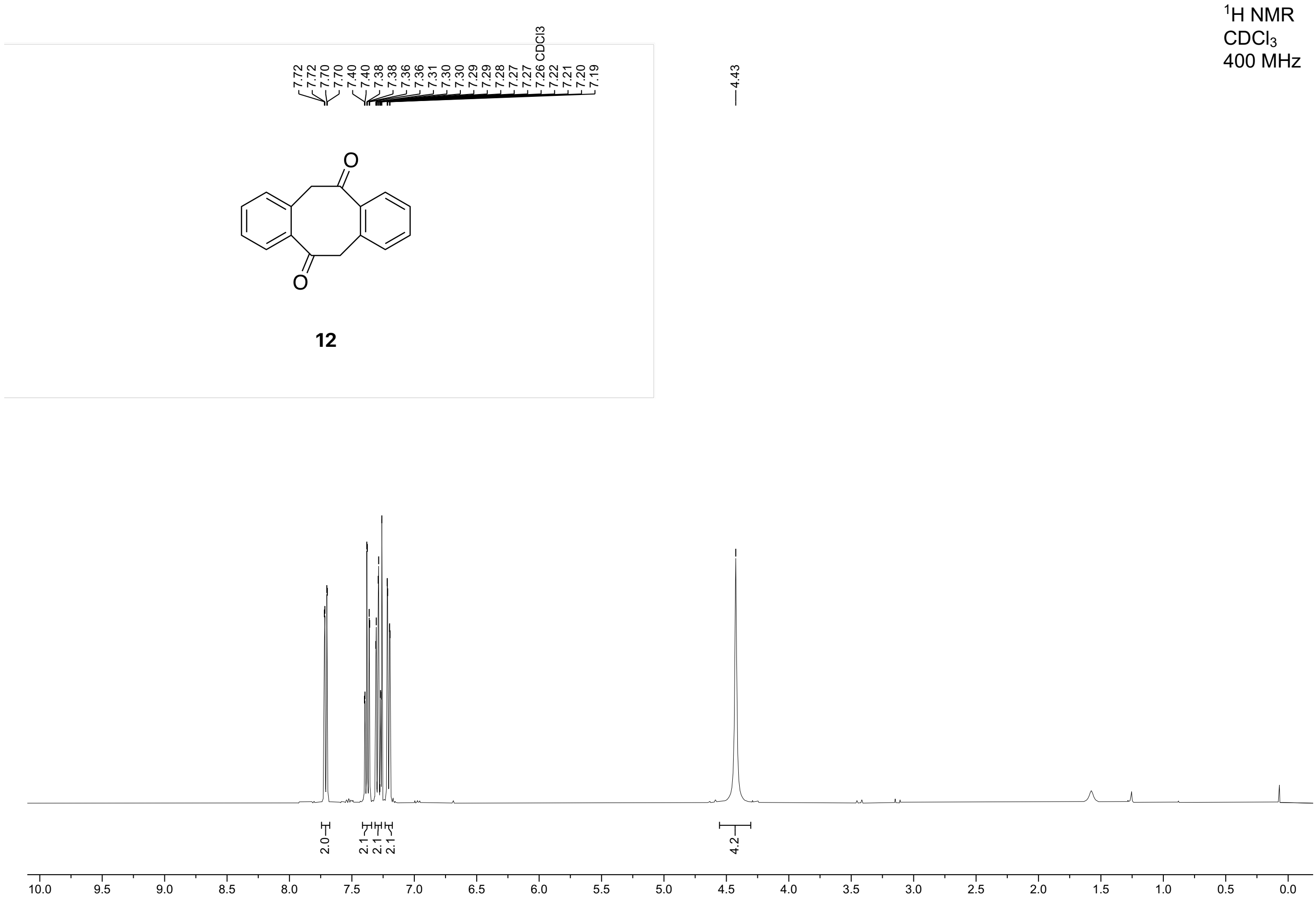

^{1}H NMR
$CDCl_3$
400 MHz
7.72 7.72 7.70 7.70 7.40 7.40 7.38 7.38 7.36 7.36 7.31 7.30 7.30 7.29 7.29 7.28 7.27 7.27 7.26 CDCl3 7.22 7.21 7.20 7.19
4.43
O
O
12
2.0 2.1 2.1 2.1 4.2
10.0 9.5 9.0 8.5 8.0 7.5 7.0 6.5 6.0 5.5 5.0 4.5 4.0 3.5 3.0 2.5 2.0 1.5 1.0 0.5 0.0

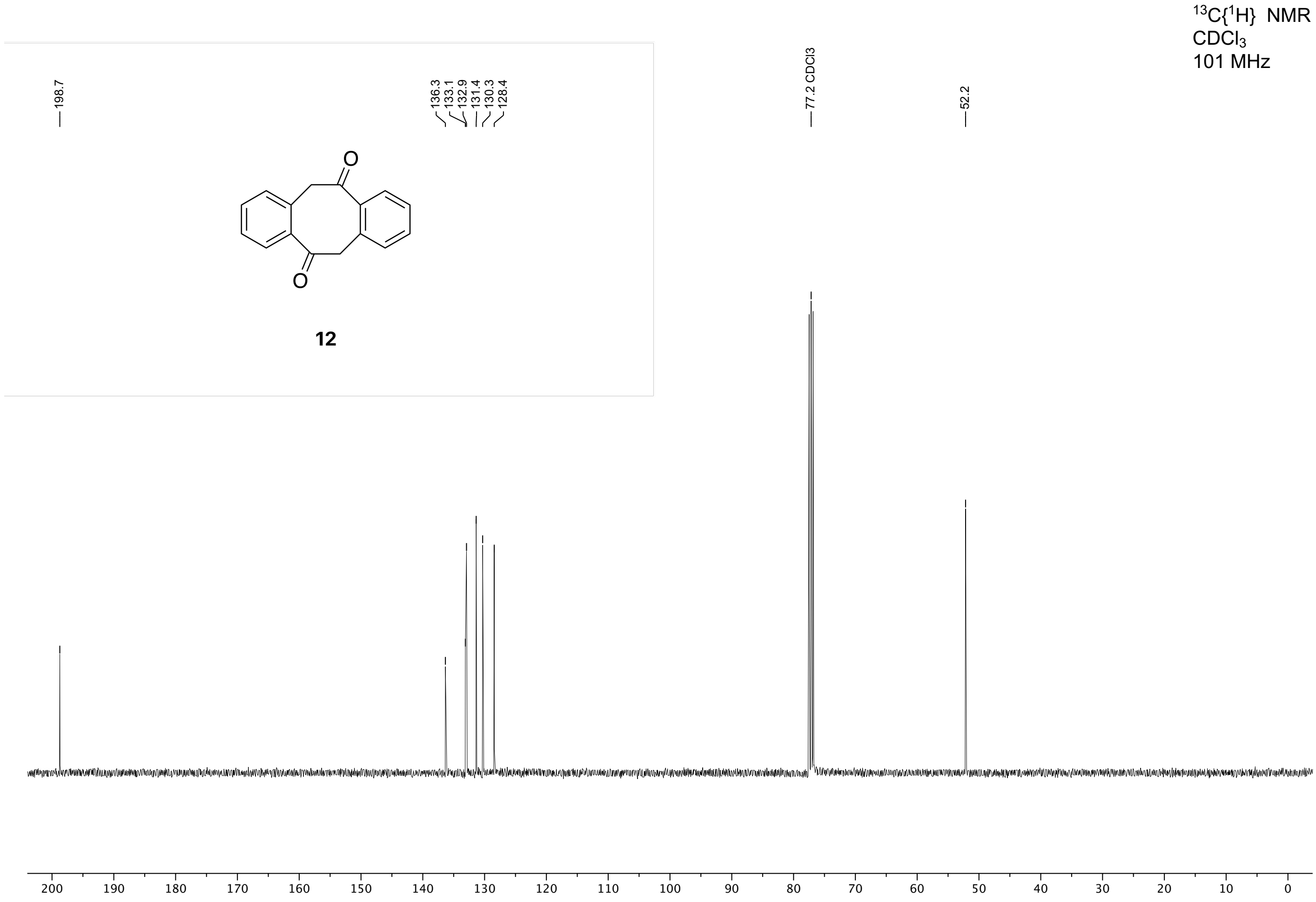

^{13}C{^{1}H} NMR
$CDCl_3$
101 MHz
198.7
136.3 133.1 132.9 131.4 130.3 128.4
77.2 CDCl3
52.2
O
O
12
200 190 180 170 160 150 140 130 120 110 100 90 80 70 60 50 40 30 20 10 0

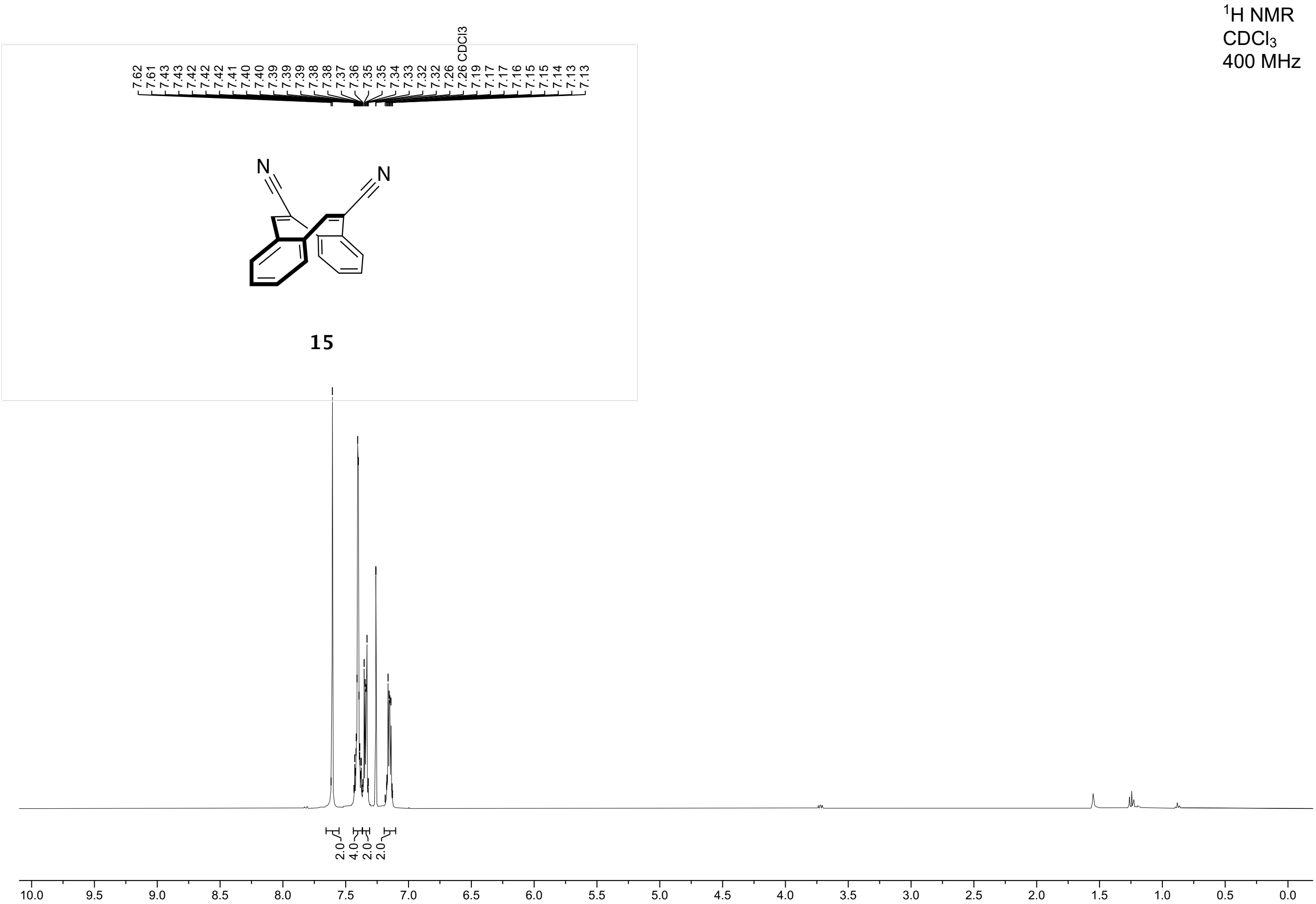

^{1}H NMR
$CDCl_3$
400 MHz
7.62 7.61 7.43 7.42 7.42 7.42 7.41 7.40 7.39 7.39 7.39 7.38 7.38 7.37 7.36 7.35 7.35 7.34 7.33 7.32 7.32 7.26 7.26 CDCl3 7.19 7.17 7.17 7.16 7.15 7.15 7.14 7.13 7.13
N
N
15
2.0 4.0 2.0 2.0

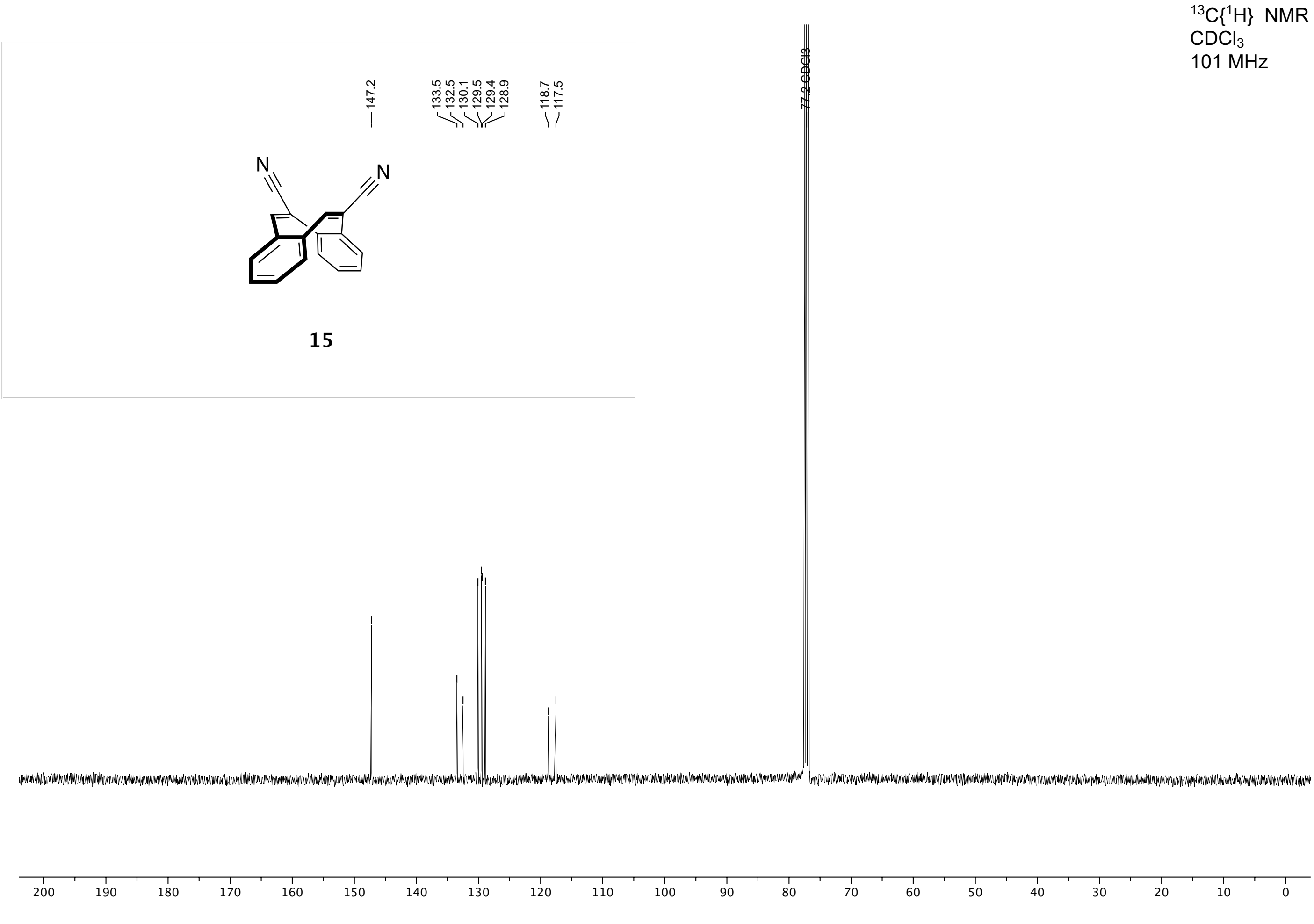

$^{13}C\{^1H\}$ NMR
$CDCl_3$
101 MHz
147.2
133.5 132.5 130.1 129.5 129.4 128.9
118.7 117.5
77.2 CDCl3
N
N
15

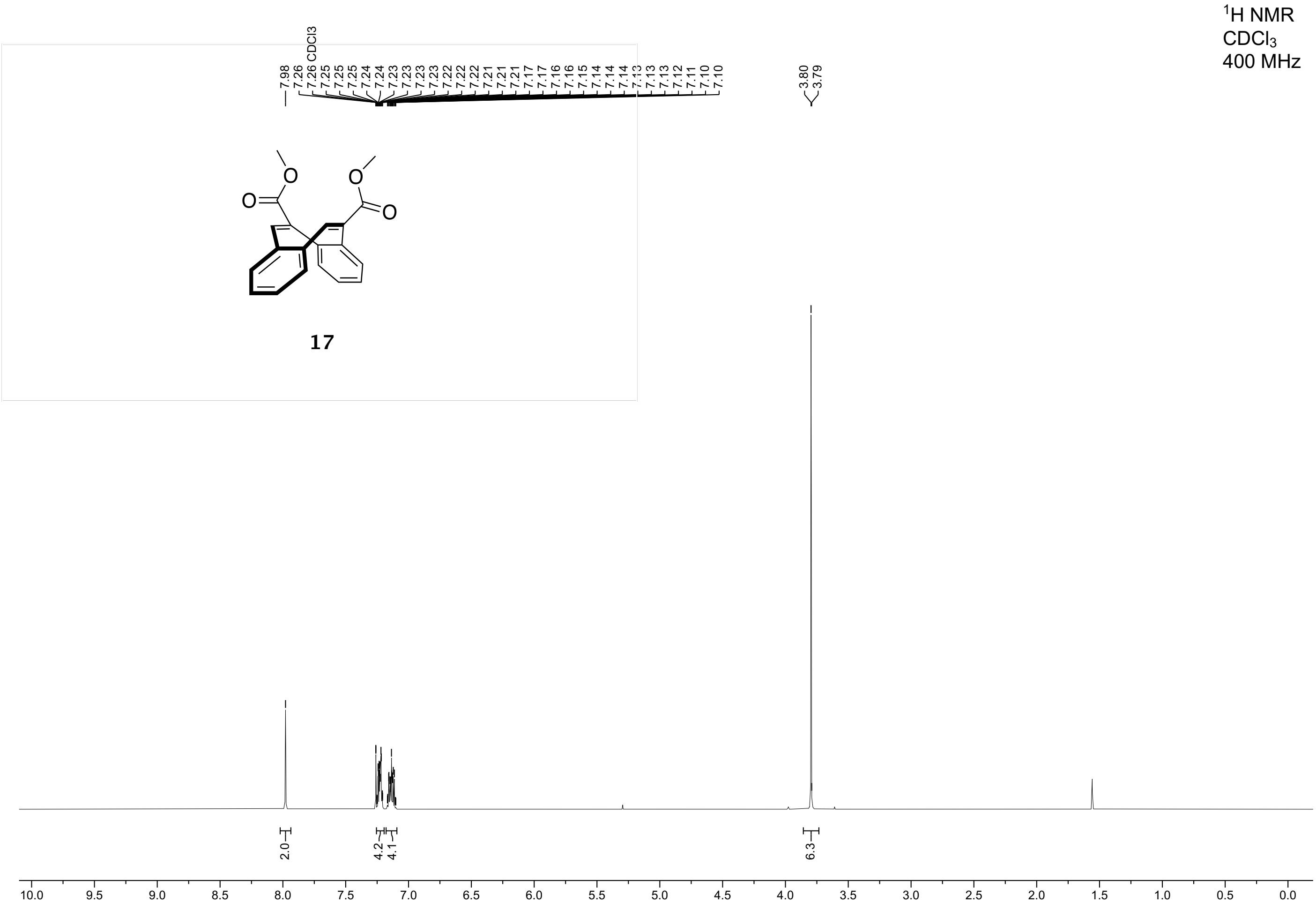
^{1}H NMR
$CDCl_3$
400 MHz
7.98
7.26 CDCl3
7.26
7.25
7.25
7.25
7.24
7.24
7.23
7.23
7.23
7.23
7.22
7.22
7.22
7.22
7.21
7.21
7.21
7.17
7.16
7.16
7.15
7.14
7.14
7.14
7.13
7.13
7.13
7.12
7.11
7.10
7.10
3.80
3.79
O
O
O
O
17
2.0
4.2
4.1
6.3
10.0 9.5 9.0 8.5 8.0 7.5 7.0 6.5 6.0 5.5 5.0 4.5 4.0 3.5 3.0 2.5 2.0 1.5 1.0 0.5 0.0

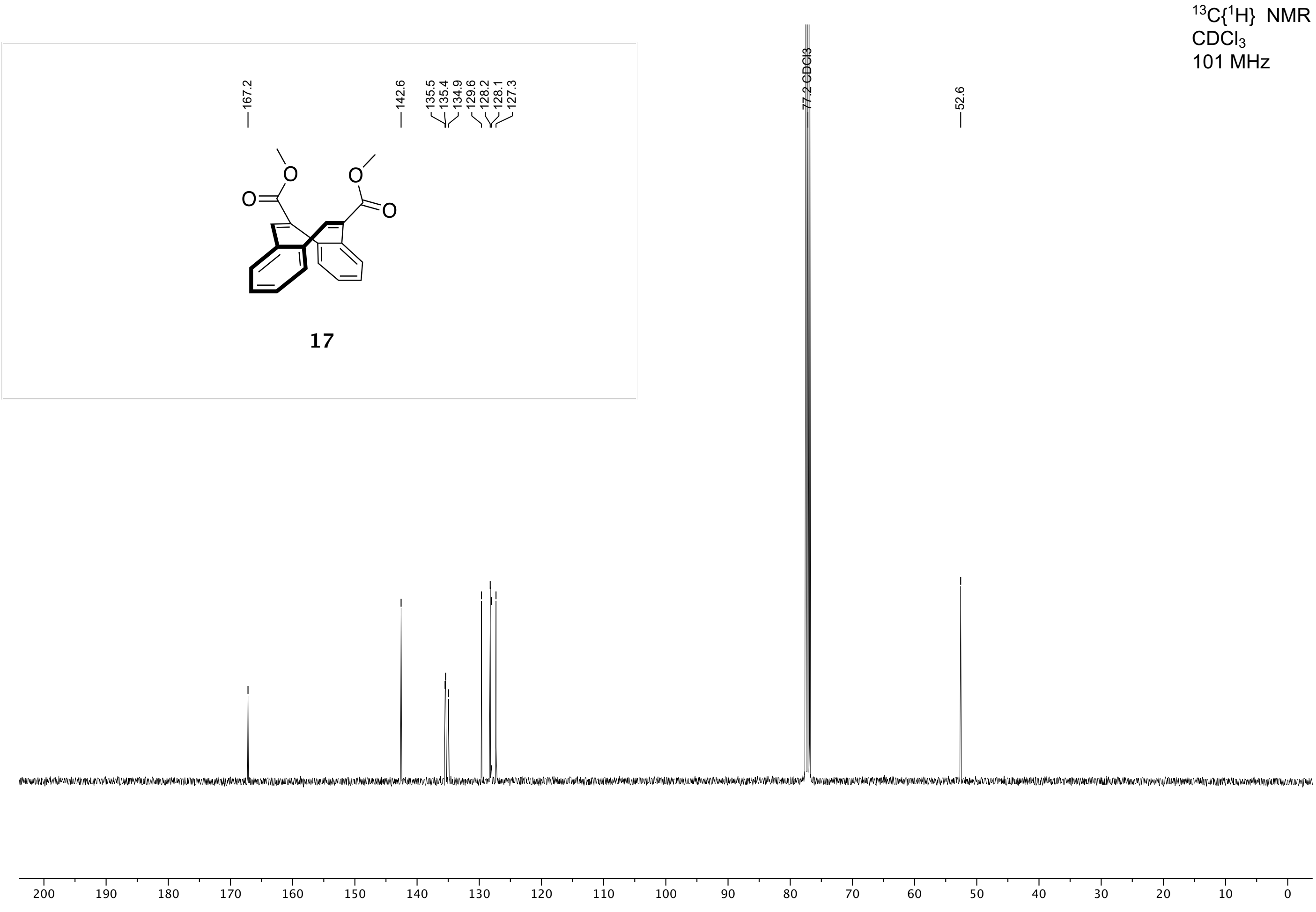
^{13}C{^{1}H} NMR
$CDCl_3$
101 MHz
167.2
142.6
135.5
135.4
134.9
129.6
128.2
128.1
127.3
77.2 CDCl3
52.6
O
O
O
O
17
200 190 180 170 160 150 140 130 120 110 100 90 80 70 60 50 40 30 20 10 0

^1H NMR
$CDCl_3$
400 MHz

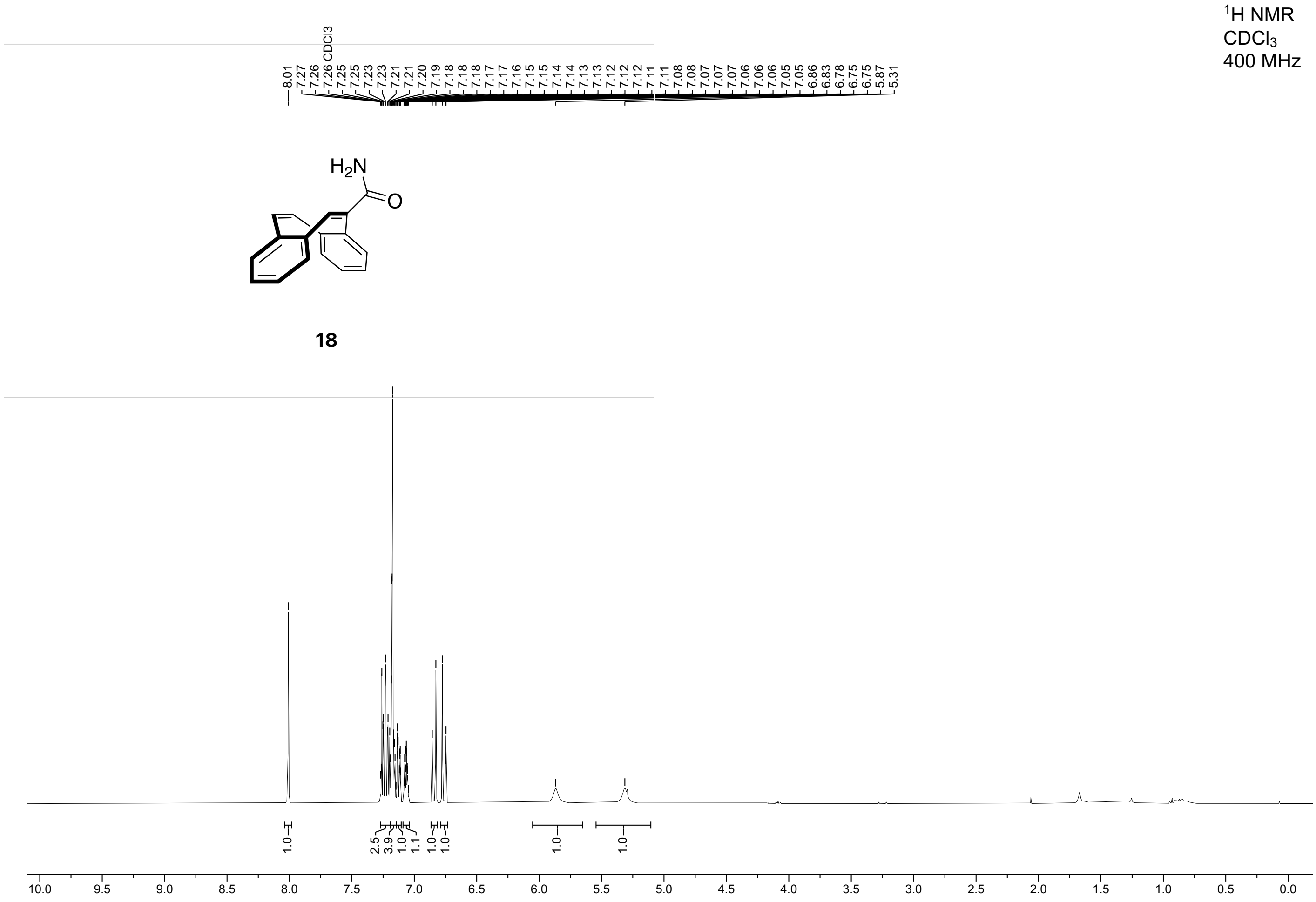

$^{13}C\{^1H\}$ NMR
$CDCl_3$
101 MHz

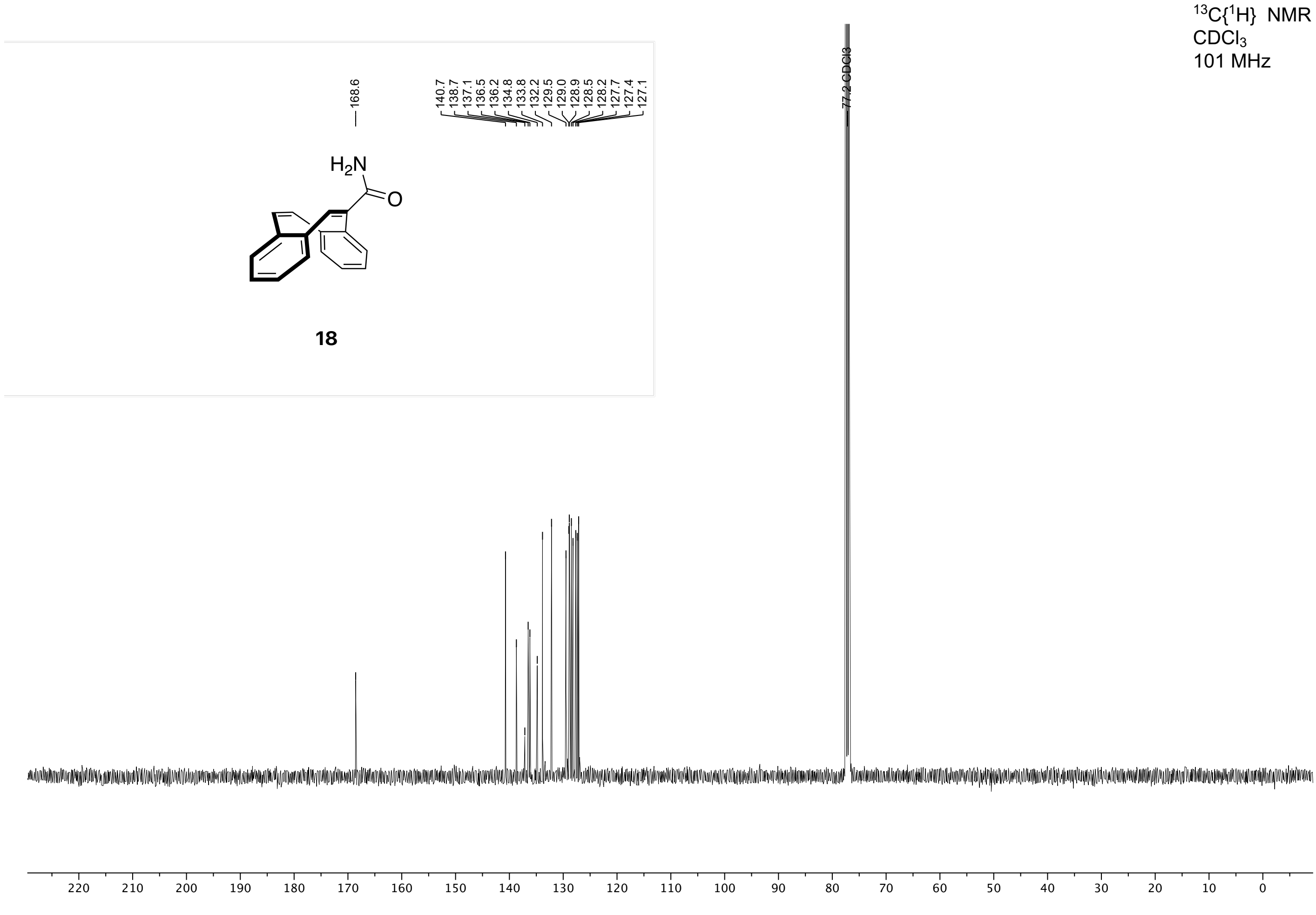

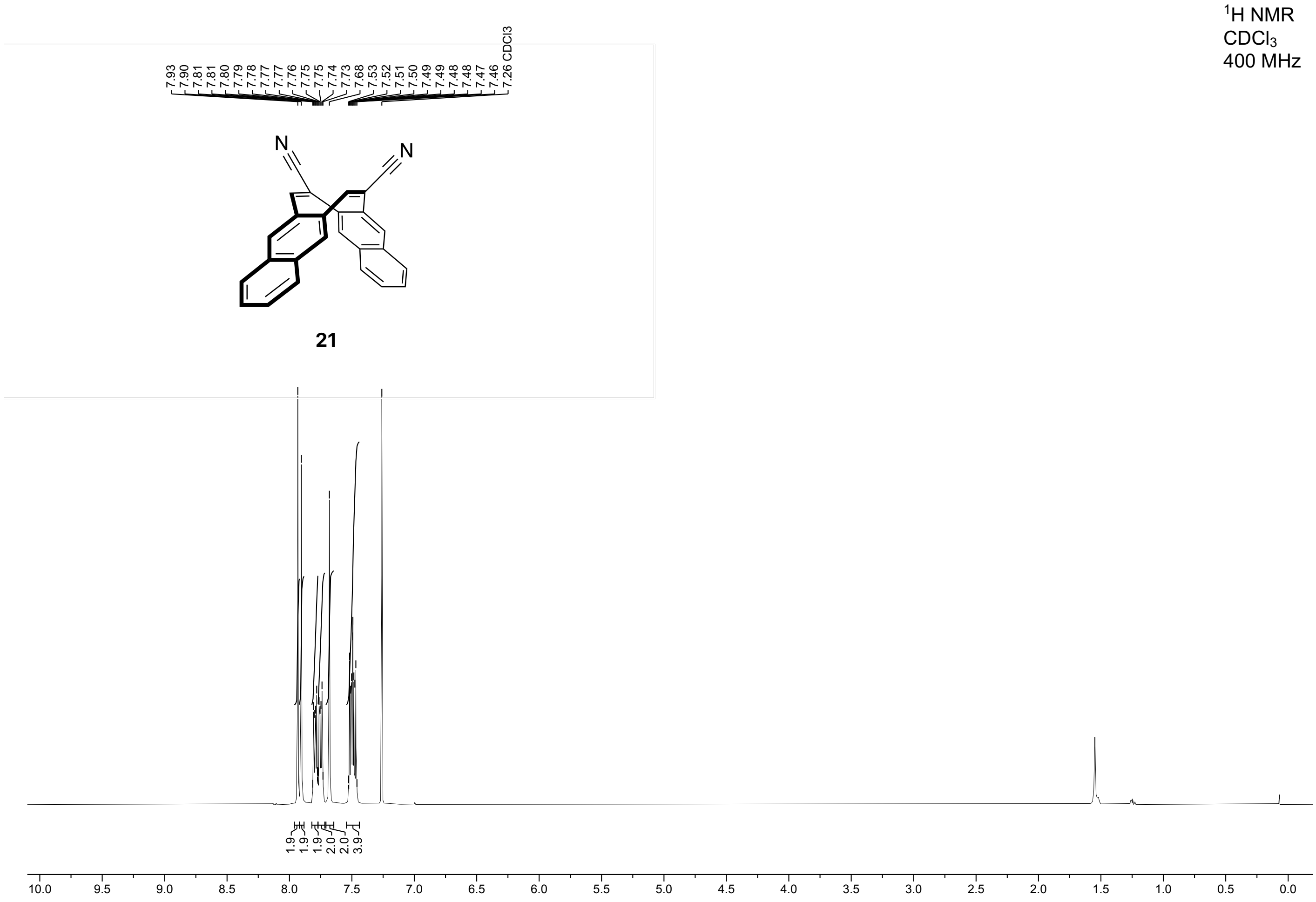

^{1}H NMR
CDCl$_3$
400 MHz
7.93 7.90 7.81 7.81 7.80 7.79 7.78 7.77 7.77 7.76 7.75 7.75 7.74 7.73 7.68 7.53 7.52 7.51 7.50 7.49 7.49 7.48 7.48 7.47 7.46 7.26 CDCl3
N
N
21
1.9 1.9 1.9 2.0 2.0 3.9
10.0 9.5 9.0 8.5 8.0 7.5 7.0 6.5 6.0 5.5 5.0 4.5 4.0 3.5 3.0 2.5 2.0 1.5 1.0 0.5 0.0

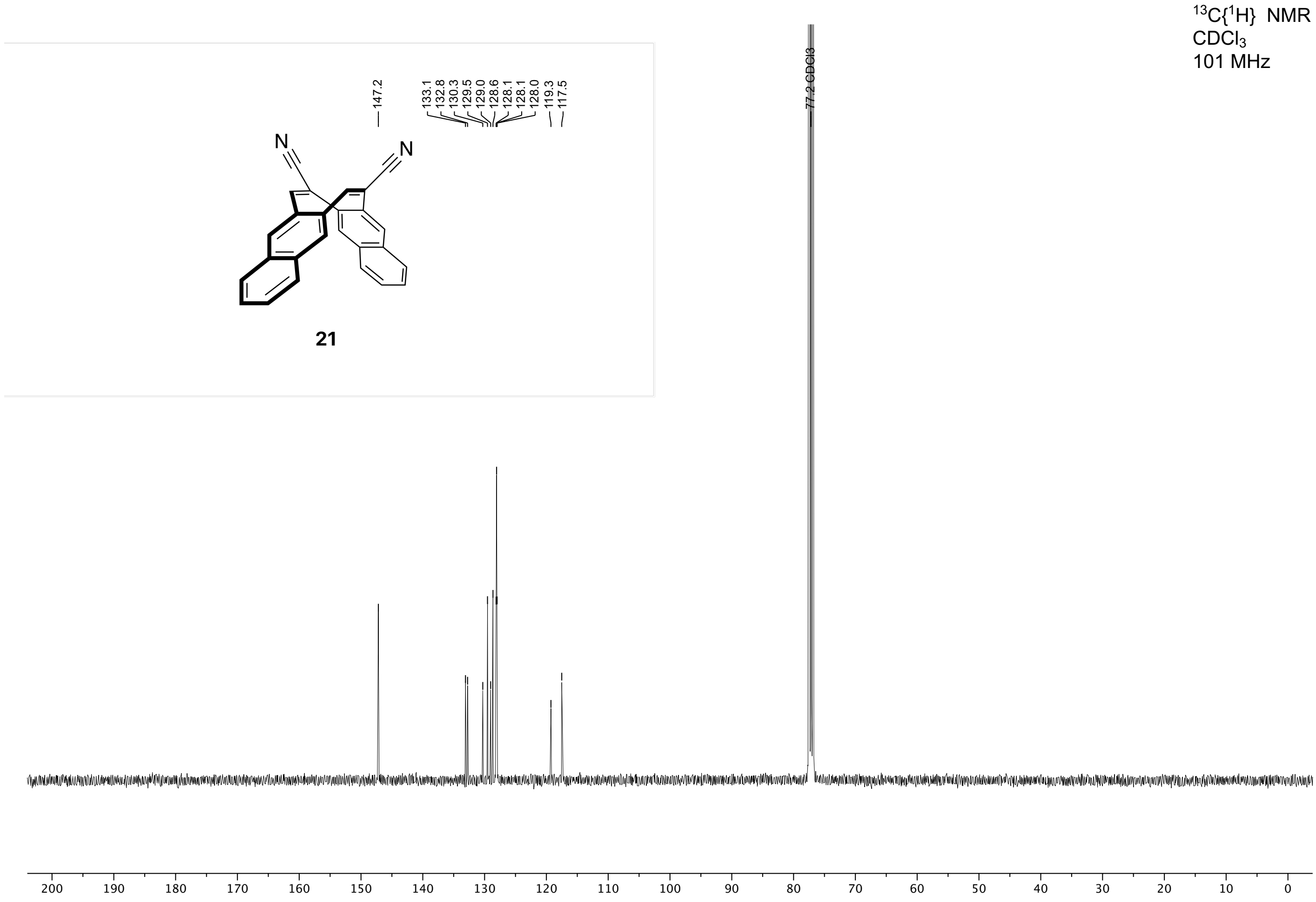

^{13}C{^{1}H} NMR
CDCl$_3$
101 MHz
147.2
133.1 132.8 130.3 129.5 129.0 128.6 128.1 128.1 128.0 119.3 117.5
77.2 CDCl3
N
N
21
200 190 180 170 160 150 140 130 120 110 100 90 80 70 60 50 40 30 20 10 0

^{1}H NMR
$CDCl_3$
400 MHz

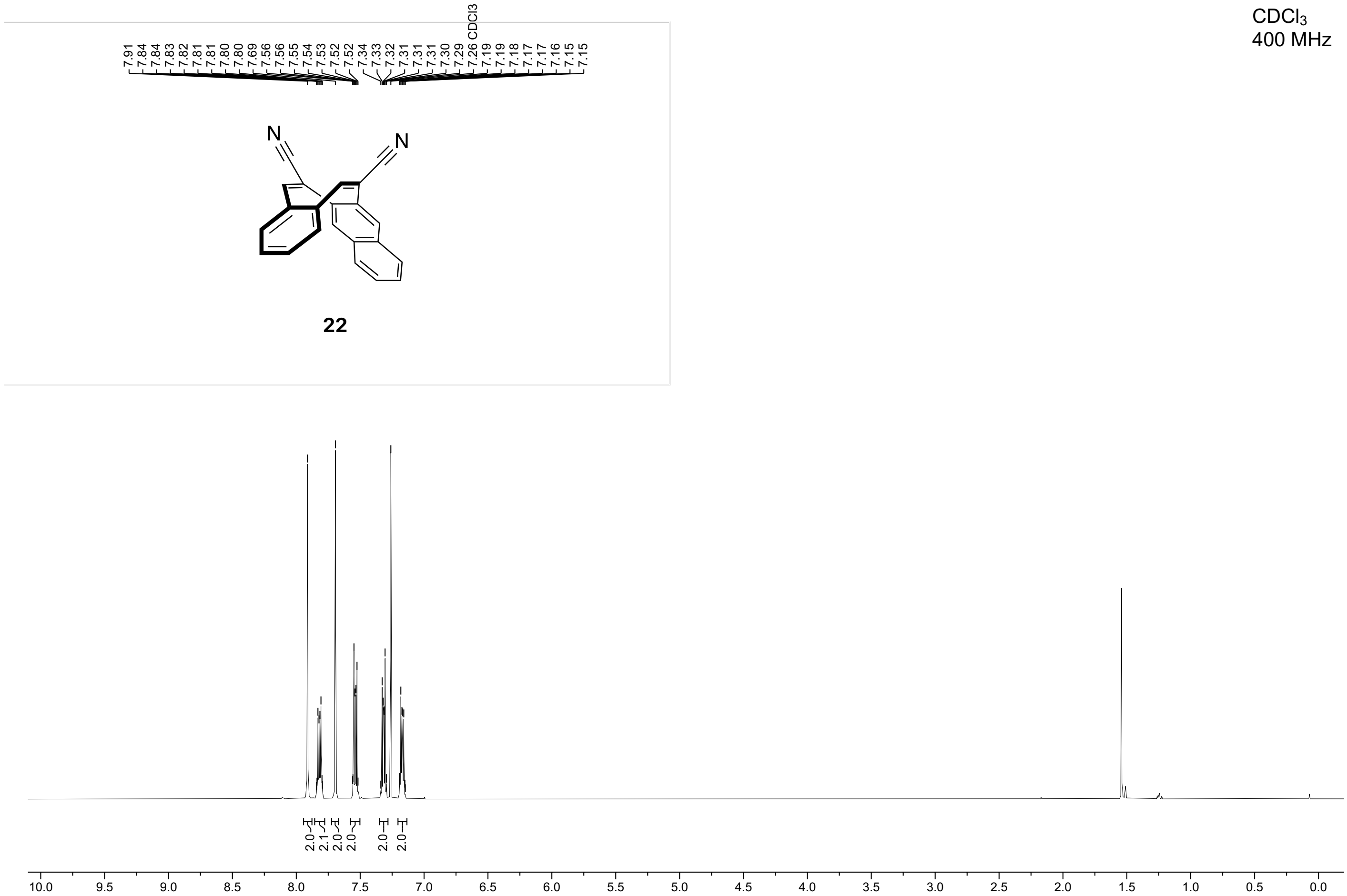

$^{13}C\{^{1}H\}$ NMR
$CDCl_3$
101 MHz

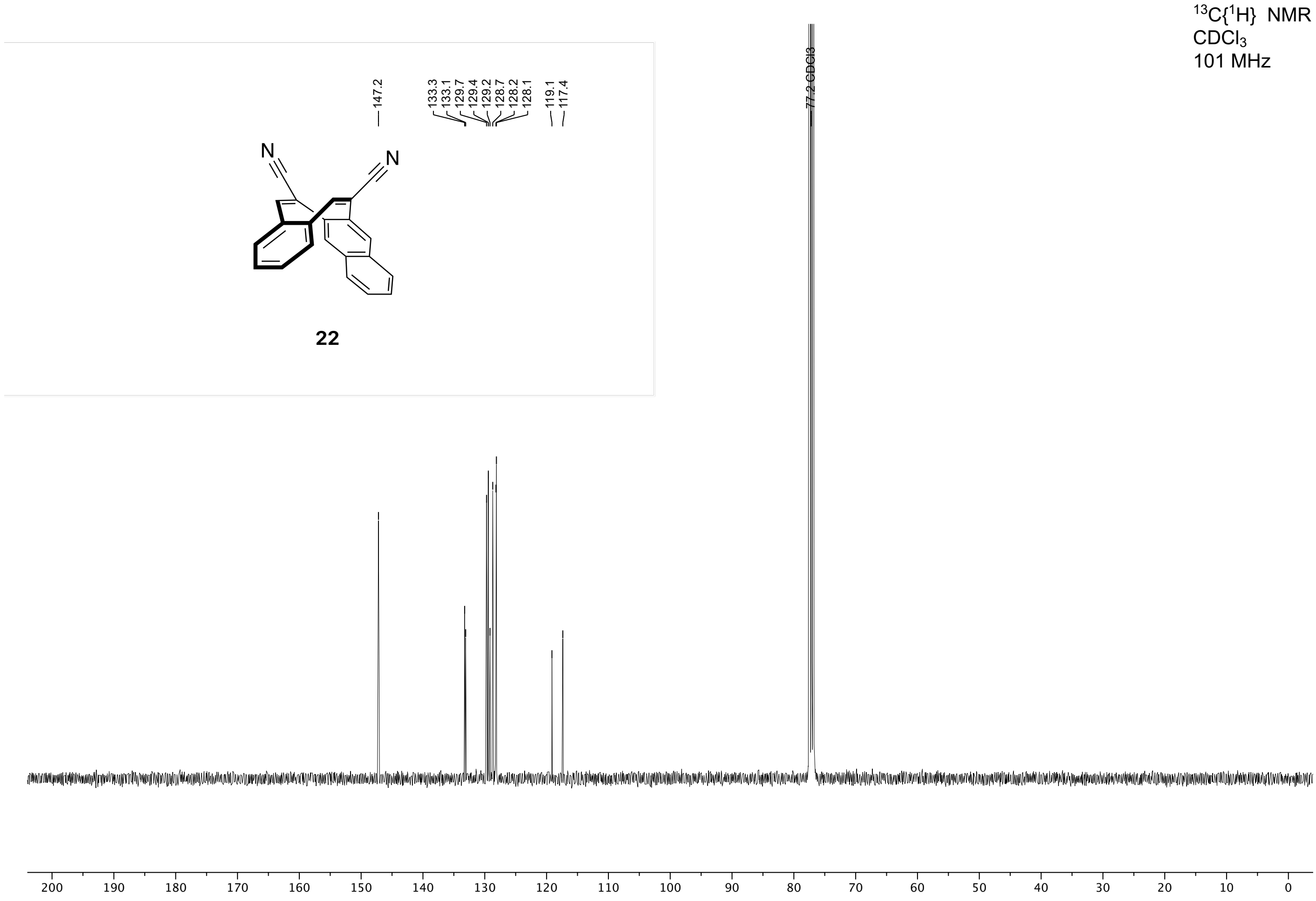

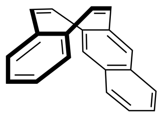

23

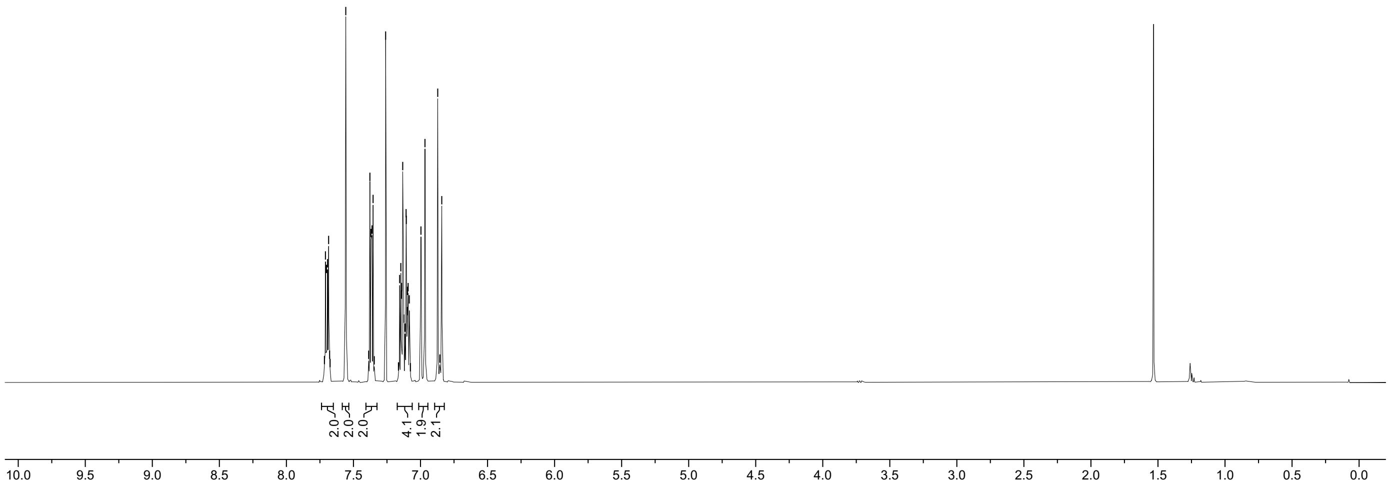
^1H NMR
$CDCl_3$
400 MHz
7.72 7.71 7.70 7.69 7.69 7.68 7.67 7.56 7.39 7.38 7.37 7.36 7.36 7.35 7.26 CDCl3 7.16 7.16 7.15 7.14 7.14 7.13 7.12 7.12 7.11 7.11 7.10 7.10 7.09 7.08 7.08 7.00 6.97 6.87 6.86 6.84
2.0 2.0 2.0 4.1 1.9 2.1
10.0 9.5 9.0 8.5 8.0 7.5 7.0 6.5 6.0 5.5 5.0 4.5 4.0 3.5 3.0 2.5 2.0 1.5 1.0 0.5 0.0

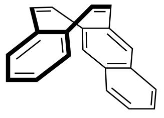

23

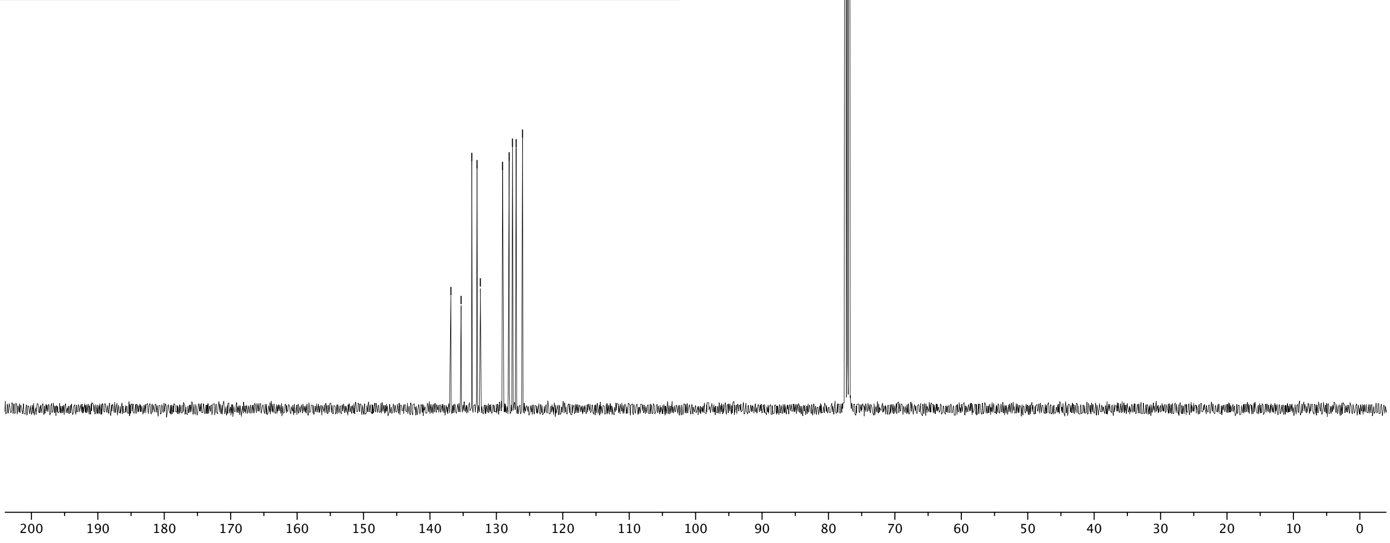
$^{13}C\{^1H\}$ NMR
$CDCl_3$
101 MHz
136.8 135.3 133.7 132.9 132.4 129.1 128.1 127.6 127.0 126.1
77.2 CDCl3
200 190 180 170 160 150 140 130 120 110 100 90 80 70 60 50 40 30 20 10 0
